# Orodispersible films containing chestnut shell phenolics for buccal delivery: a preclinical approach for oral mucositis prevention

**DOI:** 10.3389/fmedt.2025.1675082

**Published:** 2025-09-18

**Authors:** Ana Sofia Ferreira, Ana Margarida Silva, Catarina Macedo, Emily P. Laveriano-Santos, Julián Lozano-Castellón, Rosa M. Lamuela-Raventós, Jaroslava Švarc-Gajíc, Paulo C. Costa, Cristina Delerue-Matos, Francisca Rodrigues

**Affiliations:** ^1^ REQUIMTE/LAQV, ISEP. Polytechnic of Porto, Porto, Portugal; ^2^ UCIBIO—Applied Molecular Biosciences Unit, MedTech-Laboratory of Pharmaceutical Technology, Faculty of Pharmacy, University of Porto, Porto, Portugal; ^3^ Associate Laboratory i4HB – Institute for Health and Bioeconomy, Faculty of Pharmacy, University of Porto, Porto, Portugal; ^4^ Nutrition, Food Science and Gastronomy Department, School of Pharmacy and Food Science, INSA-University of Barcelona, Barcelona, Spain; ^5^ Consorcio CIBER, M.P. Fisiopatología de la Obesidad y la Nutrición (CIBERObn), Instituto de Salud Carlos III (ISCIII), Madrid, Spain; ^6^ Faculty of Technology, University of Novi Sad, Novi Sad, Serbia

**Keywords:** chestnut shells, oral mucositis, antioxidants, solvent casting, buccal *in vitro* model, porcine mucosa ex vivo assay

## Abstract

**Introduction:**

Oral mucositis (OM) is a prevalent complication of cancer treatment that causes painful erythematous and ulcerated lesions in oral mucosa. Current treatments lack efficacy, being natural compounds explored as alternatives. Chestnut shells (CS) are rich in (poly)phenols with antioxidant, anti-inflammatory, and antitumor properties. This study aims to develop orodispersible films (OFs) with CS extract as active ingredient to manage OM.

**Methods:**

OFs, prepared by solvent casting and incorporating the CS extract, were characterized regarding physicochemical, antioxidant/antiradical, and anticancer properties, as well as bioactive compounds permeation through *in vitro* and *ex vivo* buccal models.

**Results:**

OFs revealed excellent features: thickness (125 µm), tensile strength (43.05 MPa), elongation (75.28%), weight (19 mg/cm^2^), moisture content (4.25%), and disintegration time (20.43 min). Significant antioxidant/antiradical activities were observed (TPC = 37.05 mg GAE/g film; DPPH = 143.42 mg TE/g film; FRAP = 0.142 µmol FSE/g film). LC-ESI-LTQ-Orbitrap-HRMS analysis confirmed the high permeation of sebacic acid, epicatechin, isorhamnetin, protocatechuic acid, and L-tyrosine across both models, while cytotoxicity assays in oral epithelial cell lines (HSC3 and TR146) demonstrated the cytocompatibility.

**Conclusion:**

CS-OFs offers a promising approach for preventing and treating OM, enhancing efficacy and patient comfort by delivering bioactive compounds directly to the oral mucosa.

## 1 Introduction

Oral mucosa is highly sensitive to cytotoxic anticancer agents, such as chemo and/or radiotherapy, owing to their mechanism of action in rapidly targeting proliferative cell populations, including malignant cells (1, 2). These treatments induce collateral effects in healthy tissues such as the mucosal lining. The disruption of oral mucosal integrity and the generation of oxidative stress trigger an inflammatory process that result in the onset of oral mucositis (OM) (2, 3). This condition significantly impairs the patient's quality of life by causing pain, hemorrhage, ulceration, ingestion difficulties, and increased susceptibility to infections, leading to a reduction in the treatment dose or cessation, thereby adversely affecting patient prognosis (4). OM affects almost 100% of patients with head and neck cancer (HNC) subjected to radiotherapy, 70%–90% of patients undergoing hematopoietic stem cell transplantation, and up to 40% of those with solid tumors receiving chemotherapy (2, 5–7). Therefore, OM is a public health concern, particularly when 35 million new cancer cases are predicted by 2050, representing an increase of 77% when compared to 2022 (8). The current first-line treatment for OM remains inadequate, as it provides only temporary and limited pain relief. Additionally, it often leads to a greater reliance on opioid analgesics and necessitates parenteral (IV feeding) or enteral nutrition (tube feeding) for patients unable to maintain proper oral intake (9). This underscores the urgent need to explore new therapeutic approaches.

Over the last few years, natural agents have garnered significant interest due to their diverse biological properties, namely anti-inflammatory, antioxidant, antibacterial, anticancer, immunomodulatory, sedative, and healing capacities, which may represent an effective approach to prevent OM (4, 6, 10–13).

*Castanea sativa* (chestnut) shells (CS) are a widely produced by-product generated in large amounts by chestnut industry, particularly in the Southern European region, with sustainability concerns encouraging the valorization of these residues (14). Multiple studies have highlighted the abundance of (poly)phenols in CS extracts, particularly phenolic acids (gallic acid and protocatechuic acid), flavonoids (epicatechin and quercetin), and ellagitannins, along with essential vitamins and amino acids (arginine and leucine) (14–19).

This outstanding composition is responsible for different biological activities reported for CS extracts, such as antioxidant, anti-inflammatory, and antimicrobial effects, as well as gene expression regulation, enhancement of endothelial function, and enzyme inhibition (e.g., matrix metalloproteinases) (14, 18, 19). More recently, our research team attested the metabolomic profile of CS extracts using *in vitro* and *in vivo* assays in animals (mice), supporting the pro-healthy properties ascribed and opening new perspectives for its use as active ingredient to prevent OM (15, 16).

Although several biological activities have been reported for CS extracts and their phenolic constituents, these effects are often studied in different experimental contexts, using variable extraction methods, units, and dose ranges. Antioxidant and anti-inflammatory activities are consistently observed at physiologically relevant concentrations (10–100 µg/ml extract equivalents), whereas antimicrobial and antitumor effects generally require higher doses (18). Many of these bioactivities converge on common mechanisms, including modulation of oxidative stress and inflammatory signaling (e.g., suppression of TNF-α and IL-6). Importantly, reported toxicity data indicate that these concentrations are cytocompatible, which supports the relevance of focusing on antioxidant and anti-inflammatory pathways in the context of OM. The present work focused on the most mechanistically relevant and dose-feasible activities, namely antioxidant and anti-inflammatory effects, given their established role in the pathophysiology of OM. This approach provides a stronger evidence-based rationale for the potential therapeutic use of CS extracts.

Oral dosage forms continue to be the prevalent approach for drug administration owing to patient compliance and convenience, cost-effectiveness, and feasibility for large-scale manufacturing, when compared to injections and inhalers (20, 21). Nevertheless, challenges arise for geriatric and pediatric populations as well as for dysphagic patients as a consequence of OM (4). To address these limitations, orodispersible films (OFs) are gaining prominence in pharmaceutical research and development (20, 21). OFs are composite active compound-loading films formed by a polymer matrix consisting of one or more layers, with the potential to be rapidly dispersible in the mouth or mucoadhesive, leading to distinct routes of absorption (22–24). For example, active compounds that undergo rapid absorption via oral delivery avoid exposure to the gastrointestinal tract, which may suffer degradation from stomach acid, bile, and first-pass metabolism. Consequently, OFs have the potential to allow local action, reduce the required dose, and enhance the efficacy and safety profile of some active compounds (25).

This study aimed to incorporate the CS extract into OFs and evaluate their capacity to alleviate OM symptoms. To achieve this objective, the mechanical, physicochemical, antioxidant/antiradical, and cytotoxic properties of the formulated OFs were quantitatively assessed, along with the permeation of key bioactive compounds, using validated *in vitro* (buccal cell model) and *ex vivo* (porcine buccal mucosa) assays supported by appropriate statistical analysis.

## 2 Materials and methods

### 2.1 Chemicals

All chemicals and standards used were of analytical reagent grade, while all chromatographic solvents were of HPLC-HRMS grade specifications. HPMC E10M was a gift from Colorcon (USA). Glycerol, acetonitrile, formic acid, water, methanol and refence including chlorogenic acid, 2,5-dihydroxybenzoic acid, phloridzin, quercetin-3-*O*-galactoside, protocatechuic acid, *O*-coumaric acid, trans-polydatin, castalagin, neochlorogenic acid, 2,6-dihydroxybenzoic acid, gallic acid, apigenin, luteolin, naringin, rutin, 3-hydroxyphenylacetic acid, 3,5-di-caffeoylquinic acid, dihydroxyphenylpropionic acid, 3-hydroxybenzoic acid, epicatechin, p-coumaric acid, ferulic acid, secoisolariciresinol, isorhamnetin, dihydroferulic acid, vanillin, and catechin, were obtained from Sigma-Aldrich (Steinheim, Germany). Methyl gallate was supplied by Phytolab (Vestenbergsgreuth, Germany). Human tongue squamous cell carcinoma (HSC-3), human squamous cell carcinoma (TR146), and the human epidermal keratinocyte cell line (HaCaT) were obtained from the American Type Culture Collection (ATCC, USA). Cell reagents were purchased from Invitrogen Corporation (Life Technologies, S.A., Madrid, Spain).

### 2.2 *Castanea sativa* shells extract

*Castanea sativa* shells were kindly supplied by Sortegel (Sortes, Bragança, Portugal). After being dehydrated and shredded, the samples underwent subcritical water extraction (SWE) following the methodology outlined by Ferreira et al. (14). In summary, the extraction was carried out at 110°C using a custom-built subcritical batch-type extractor (1.7 L) equipped with a built-in valve and pressurized with 99.99% pure nitrogen (Messer). The process lasted 30 min at a pressure of 20 bar, maintaining a sample-to-solvent ratio of 1:30. The extraction vessel was agitated on a vibrating platform (3 Hz) and subsequently cooled in a water bath (20 ± 2°C) with continuous flow (14). Following extraction, the liquid extract was centrifuged at 11,000 rpm for 10 min to eliminate any solid residues before being incorporated into the OFs.

### 2.3 Preparation of OFs

Preliminary experiments using the solvent casting method were conducted to identify the optimal OFs polymers and their respective concentrations suitable for the intended applications (26). Similarly, the CS concentration was determined through preliminary testing to establish the maximum concentration that allowed to produce OFs with uniform content. Following optimization, HPMC E10M was used at a concentration of 1% (*w/v*), whereas glycerin was employed as a plasticizer at 2.5% (*w/v*). Briefly, the polymer and glycerin were dispersed in 100 ml of deionized water for the placebo OFs. For the OFs incorporating the CS extract (CS-loaded OFs), the liquid form of the extract was added to the mixture at a concentration of 25% (*v/v*) as solvent (∼0.25 g of dry extract per g film (*w/w*)). The resulting solution was subsequently spread onto a plastic petri dish (8.5 cm) and refrigerated overnight at 4°C, to remove any entrapped air. The following day, the solvent evaporation was controlled by drying the films in an oven at 60°C for 24 h under covered conditions, followed by storage in a desiccator to ensure consistent drying and minimize solvent loss.

### 2.4 Characterization of OFs

#### 2.4.1 Thickness and weight uniformity

The films thickness (*n* = 9) were measured on conditioned samples, which were equilibrated at 50 ± 5% relative humidity (RH) and 23 ± 2°C for 48 h prior to measurement to ensure consistent and representative results. Films were cut into 2 × 2 cm squares, and the thickness was determined using a digital micrometer (Powerfix Z22855, Germany) at three distinct points. The films weight were determined by cutting 2 × 2 cm squares (area = 4 cm^2^) and weighing them individually (*n* = 3) using an analytical balance (Radwag AS 220.R2, Poland). For standardization, and to account for possible differences in portion size and thickness uniformity, the results were expressed as weight per unit area (mg/cm^2^), obtained by dividing the measured film weight (mg) by the specimen area (4 cm^2^). To ensure reproducibility and comparability, the methodology applied for film preparation and testing was aligned with the ISO 37:2017 standard that establishes standardized specimen dimensions and procedures for evaluating thin films.

#### 2.4.2 Mechanical properties

Mechanical properties were measured on conditioned samples (*n* = 9), equilibrated at 50 ± 5% RH and 23 ± 2°C for 48 h. Films were cut into rectangles measuring 1 × 5 cm, and their mechanical properties were determined using a texture analyzer (TA.XT plus Texture Analyzer, Stable Micro Systems, Cardiff, UK) with Miniature Tensile Grips (Stable Micro Systems). Data were collected using Texture Exponent 32 software (version 6.1.12.0; Stable Micro Systems, Surrey, UK). Three independent film portions (*n* = 3) were held vertically with a separation of 10 mm and stretched until rupture by moving the probe at a constant speed of 0.1 mm/s. The tensile strength (N), elongation at break (%), and Young's modulus (MPa) were determined from the stress-strain curves as follows:

$$\begin{array}{rl} & \mathrm{Elongation}\;(\text{\%})=\\ & \frac{\mathrm{distance}\;\mathrm{at}\;\;\mathrm{the}\;\;\mathrm{rupture}\;\mathrm{instant}\text{-}\mathrm{inicial}\;\;\mathrm{grip}\;\mathrm{distance}}{\mathrm{inicial}\;\mathrm{grip}\;\mathrm{distance}}\;\times 100\text{\%} \end{array}$$

$$\begin{array}{rl} & \mathrm{Youn}g^{\prime }s\;\;\mathrm{modulus}\;\;\left(\mathrm{MPa}\right)=\\ & \frac{\mathrm{force}\;\mathrm{at}\;\mathrm{corresponding}\;\mathrm{strain}}{\mathrm{cross}\text{-}\mathrm{sectional}\;\mathrm{area}\;\;\mathrm{of}\;\mathrm{film}\times \mathrm{corresponding}\;\mathrm{strain}} \end{array}$$

#### 2.4.3 Folding endurance

Films (*n* = 9) were cut into equal sizes (2 × 2 cm) and folded repeatedly until breakage at some point or completion of a maximum of 300 folds, which is considered an excellent flexibility ability (26). Similarly to the previous assays, the films were previously equilibrated at 50 ± 5% RH and 23 ± 2°C for 48 h.

#### 2.4.4 Surface pH

Films (*n* = 9; 2 × 2 cm) were immersed in a Petri dish filled with artificial saliva (pH 6.8) for approximately 1 min. Artificial saliva was prepared according to the method described by Hobbs et al. (27) and kept at 37 ± 1°C. A pH meter S400 (Mettler-Toledo, Ohio, USA) electrode was placed on the film surface, and pH readings were recorded.

#### 2.4.5 Swelling capacity

The hydration capacity of the films (*n* = 9; 2 × 2 cm) was measured by weighing the samples over time during contact with artificial saliva. Each film was weighed (W1), placed onto a glass Petri dish containing 3 ml of artificial saliva, removed after 5 min, and reweighed (W2). The swelling index was calculated as follows:

$$\mathrm{Swelling}\;\mathrm{capacity}\;\left(\text{\%}\right)=\frac{W2-W1}{W1}\;\times 100$$

#### 2.4.6 Moisture content

Films (*n* = 9; 2 × 2 cm) were placed in an infrared moisture balance AD-4713 (A&D Company, Japan) at 100°C for 20 min. The device autonomously determined the moisture percentage by analyzing the difference in the films weight before and after heating.

#### 2.4.7 Disintegration time

The disintegration time of the formulated films (*n* = 9; 2 × 2 cm) was assessed using the petri dish method (26). Briefly, 10 ml of artificial saliva was placed in a glass petri dish and the temperature was kept constant at 37 ± 1°C. Films were then introduced into the petri dishes and subjected to rotation at 50 rpm using a magnetic stirrer (IKA C-MAG HS7, Carl-Roth, Germany), measuring the time taken by them to be completely disintegrated.

#### 2.4.8 Stability tests

Films (*n* = 9; 2 × 2 cm) were subjected to accelerated stability tests by storage at 40°C with 75% relative humidity (40°C/75% RH) and 25°C with 65% RH (25°C/65% RH) and wrapped in aluminum foil for 90 days (28). Subsequently, the tensile strength, elongation, folding endurance, surface pH, and disintegration time were evaluated at time 0 and after 15, 30, 60, and 90 days of storage under both conditions.

#### 2.4.9 Total phenolic content

Total Phenolic Content (TPC) was assessed by spectrophotometry following the Folin–Ciocalteu method, with minor changes (14). Two square films (*n* = 9; 2 × 2 cm) were dissolved in 4 ml of artificial saliva to create a 100% film-concentrated stock solution, from which serial dilutions were prepared. A calibration curve (linearity range: 5–100 µg/ml; *R*^2^ > 0.997) was established using gallic acid as the standard. The results are presented as milligrams of Gallic Acid Equivalents (GAE) per gram of film (mg GAE/g film).

#### 2.4.10 DPPH assay

The DPPH free radical-scavenging assay was performed according to the protocol described by Pinto et al. (19). Trolox was used as standard for the calibration curve (linearity range: 5–125 µg/ml; *R*^2^ > 0.996). Films (*n* = 9; 2 × 2 cm) were treated as described in Section 2.4.9. The results are presented as milligrams of Trolox Equivalents (TE) per gram of film (mg TE/g film).

#### 2.4.11 FRAP assay

FRAP assay was performed as described by Ferreira et al. (14). A calibration curve (linearity range: 25–500 µM; *R*^2^ > 0.998) was established using a standard ferrous sulfate (FeSO_4_ · 7H_2_O) solution at a concentration of 1 mM. Films (*n* = 9; 2 × 2 cm) were treated as described in Section 2.4.9. The results are expressed in µmol of ferrous sulfate equivalents (FSE) per gram of film (µmol FSE/g film).

#### 2.4.12 Scanning electron microscopy (SEM)

SEM analysis was performed using a high-resolution (Schottky) Environmental Scanning Electron Microscope with x-Ray Microanalysis and Electron Backscattered Diffraction (FEI Quanta 400 FEG ESEM/EDAX Genesis X4M). Samples were coated with an Au/Pb thin film for 80 s and with a 15 mA current by sputtering using SPI Module Sputter Coater equipment.

#### 2.4.13 Fourier transform infrared spectroscopy (FTIR)

The interactions between the OFs polymeric matrix and the incorporated extract were evaluated using an FTIR Nicolet 6700—Diamond Point (Thermo Fisher Scientific, USA) and the potassium bromide (KBr) method (29). Samples were individually placed in the sampler with spectral analysis between 4,000 and 400 cm^−1^ and 32 scans at a resolution of 4 cm^−1^.

#### 2.4.14 Thermal properties (DSC)

Differential scanning calorimetry (DSC) thermograms of the OFs and lyophilized chestnut shells extract were obtained using a DSC 200 F3 Maia (Netzsh-Geratebau GmbH, Germany) with an empty aluminum pan as a reference. 5–10 mg of samples were placed in a sealed aluminum pan and heated from 0 to 200°C at a ramping rate of 10°C/min. Nitrogen was used as the purging gas at a flow rate of 20 ml/min. The onset temperatures were calculated using the Proteus Analysis software (version 6.1, Netzsh-Geratebau GmbH, Germany).

#### 2.4.15 Mucoadhesive strength

Porcine buccal mucosal tissue was obtained from a local slaughterhouse to evaluate the mucoadhesive properties of the prepared films. Using a texture analyzer coupled with a mucoadhesion rig (A/MUC) from Stable Micro Systems (30), 2 cm^2^ of buccal tissue with a thickness of 500 mm ± 100 mm was hydrated for 10 min using artificial saliva and fixed in the apparatus. Films were attached to a probe with a diameter of 10 mm. Afterwards, films (*n* = 9) were in contact with the mucosa tissue by applying a downward force of 0.5 N for 30 s before conducting the experiment. The probe was raised at a constant speed of 0.3 mm/s, and the force required for complete detachment (N) and work of adhesion (N/mm) was calculated using Exponent software.

### 2.5 Cytotoxicity

OFs cell viability was assessed using an MTT assay in two human cancer cell lines, namely HSC3 and TR146, as well as in an immortalized human keratinocyte cell line, HaCaT. Passages 18, 33, and 39 were used for HSC3, TR146, and HaCaT cells, respectively. Cells were cultured and plated as described by Ferreira et al. (14). OFs were serially diluted (3%–50%) from the stock solution (100%) in DMEM. Films (2 × 2 cm) were dissolved in 10 ml of DMEM as a 100% solution. Briefly, cells (2.5 × 10^4^ cells per ml) were incubated during 24 h with fresh medium in the absence or presence of the samples. Following the samples removal from each well, cells were washed with HBSS. The number of viable cells was determined by adding MTT reagent and incubating for 3 h at 37°C. DMSO was used to solubilize the crystals. The positive control used was DMEM and the negative control was 1% (*w/v*) Triton X-100. Cell viability results are expressed as percentages (%).

### 2.6 *In vitro* permeation

The *in vitro* permeability of the bioactive compounds present in OFs (*n* = 9) was determined using a co-culture model composed of TR146 (31). The extract and the CS-loaded OFs were added to the apical side of the model as a stock solution. Samples from the basolateral side were collected at different timepoints (0, 15, 30, 45, 60, 90, 120, 150, 180, and 240 min) and subsequently analyzed by LC-ESI-LTQ-Orbitrap-HRMS (Section 2.8). The Transepithelial Electrical Resistance (TEER) of the model was evaluated before, during, and at the end of the permeability assay using an EVOM Epithelial Volthometer equipped with a chopstick electrode (World Precision Instruments, Sarasota, FL, USA).

### 2.7 *Ex vivo* permeation

Porcine buccal mucosa was used to evaluate the buccal permeation of the phenolic compounds in the extract and the CS-loaded OFs (*n* = 9). The porcine buccal mucosa was purchased in a local butcher shop, not requiring ethical approval. A Franz cell assembly (9 mm clear jacketed Franz cell with a flat ground joint, 5 ml receptor volume, and permeation area of 0.785 cm^2^; PermeGear, Inc., USA) was used. This experiment followed the methodology described by Rodrigues et al. (32). In the Franz apparatus, the porcine buccal mucosa was positioned with the cheek side facing the donor chamber, which contained 500 µl (1,000 μg/ml) of the CS extract or the CS-loaded OFs (two square films (2 × 2 cm) with 500 ± 100 mm of thickness were dissolved in 4 ml of artificial saliva; donor concentration was normalized to ensure equivalence between the extract solution and the film formulation). The receptor chamber was filled with 5 ml PBS, maintained at 37°C, and stirred continuously at 150 rpm. The volume was maintained through the experiments. Samples (300 µl) were collected at specific timepoints (0, 15, 30, 45, 60, 90, 120, 150, 180, 210, 240, 300, 360, 420, and 480 min) and analyzed using LC-ESI-LTQ-Orbitrap-MS (Section 2.8) to determine the amount of phenolic compounds that permeated the buccal mucosa.

### 2.8 Metabolomic profile by LC-ESI-LTQ-orbitrap-HRMS

The identification and quantification of the phenolic compounds present in the extract and the OFs, as well as the compounds that permeated from the OFs through *in vitro* and *ex vivo* assays, were conducted using an LC-ESI-LTQ-Orbitrap-HRMS equipment with an Accela chromatograph (Thermo Scientific, Hemel Hempstead, UK), a photodiode array detector, a quaternary pump, and a temperature-controlled autosampler coupled to a high-resolution LTQ Orbitrap Velos mass spectrometer (Thermo Scientific, Hemel Hempstead, UK) with an ESI source in negative mode (33, 34). The system was controlled using the Xcalibur v3.0 software (ThermoFisher Scientific, Hemel Hempstead, UK). Elution was performed on an Acquity™ UPLC® BEH C18 Column (2.1 × 100 mm, i.d., 1.7 µm particle size, Waters Corporation, Wexford, Ireland) maintained at 30°C.

Gradient elution was performed with water (A) and acetonitrile (B), both with 0.1% formic acid, with a flow rate and injection volume of 450 µl/min and 5 µl, respectively. The solvent gradient (*v/v*) of B [t (min), %B] was set as follows: (0, 0), (2, 0), (4, 30), (8, 100), (10, 100), (11, 0), and (14, 0). The samples were analyzed in the full scan mode at a resolving power of 30,000 and *m/z* 600. Data-dependent MS/MS events were acquired at a resolution of 15,000. Most intense ions were detected via the FTMS mode-triggered data-dependent acquisition mode. Ions that were not sufficiently intense for a data-dependent scan were explored in MSn mode. Precursors were fragmented by collision-induced dissociation using a C-trap with a normalized collision energy (35 V) and an activation time of 10 ms. Operation parameters were as follows: source voltage, 3 kV; sheath gas, 50 units; auxiliary gas, 20 units; sweep gas, 2 units, and capillary temperature, 375°C (33). Compounds whose theoretical [M–H]^−^ values exceeded the MS¹ acquisition range (*m/z* 100–600) were annotated based on characteristic MSⁿ fragment ions detected within the scan window. These fragment-based identifications (e.g., rutin and verbascose) were considered putative.

Compounds identified were putatively annotated using the MS-finder and MS-dial software (open source version 4.25, created by Prof. Masanori Arita team (RIKEN) and Prof. Oliver Fiehn team (UC Davis)) (35–37), for data treatment, considering the high confidence provided by the fragmentation pattern, isotopic pattern (isotopic spacing and isotopic ratio) followed by exact mass and retention time alignments. A database set by combining annotations from Phenol-Explorer (http://phenol-explorer.eu/ (accessed on 27 October 2023)) and Food Database (http://foodb.ca/ (accessed on 27 October 2023)) was employed as a reference for putative annotation.

Quantitative analysis was performed by using a validated chromatographic method (38). The calibration curves (0.05–1 ppm) were as follows:

*Gallic acid:* y = −859.209 + 15446x (*R*^2^ = 0.9989)
*3-Hydroxybenzoic acid:* y = 2646.83 + 226224x (*R*^2^ = 0.9918)
*2,5-Dihydroxybenzoic acid:* y = −7872.05 + 387539x (*R*^2^ = 0.9979)
*Dihydroferulic acid*: y = −1597.01 + 39126x (*R*^2^ = 0.9953)
*Epicatechin:* y = −34724.6 + 684895x (*R*^2^ = 0.9912)
*Chlorogenic acid:* y = −25845.1 + 622416x (*R*^2^ = 0.9925)
*Phlorizin:* y = 1349.6 + 188575x (*R*^2^ = 0.9939)
*Naringin:* y = −424.528 + 572027x (*R*^2^ = 0.9975)
*Rutin:* y = −12789.3 + 365370x (*R*^2^ = 0.9945)
*Protocatechuic acid:* y = −9322.36 + 255908x (*R*^2^ = 0.9917)

The results were expressed as the permeation (%) percentage of each compound in the *in vitro* and *ex vivo* buccal models.

### 2.9 Statistical analysis

Results are expressed as the mean ± standard deviation from at least three independent experiments. Statistical analysis was performed using one-way ANOVA, following verification of normality and homogeneity of variances with the Shapiro–Wilk and Levene's tests, respectively. Tukey's HSD test was used for *post hoc* multiple comparisons. All analyses were conducted using IBM SPSS Statistics 28.0 software (Chicago, IL, USA), and differences were considered statistically significant at *p* < 0.05.

## 3 Results and discussion

### 3.1 Preparation of OFs

In addition to their resistance and stability, OFs must be manageable and flexible. Moreover, OFs must have adequate mucoadhesive properties and release the bioactive compounds, allowing the permeation through the buccal mucosa. The OFs polymers used in the present study were selected after conducting a literature review and obtaining preliminary results (data not shown). Based on qualitative parameters such as flexibility and uniformity, HPMC E10M, a semi-synthetic polymer derived from cellulose, was selected. The application of cellulosic polymers in the production of solid extended-release dosage forms is facilitated by their exceptional film-forming properties and extensive grade selection, despite not being pH-responsive (39). At 2% (water) and 20°C, HPMC E10M, a medium molecular weight HPMC, possesses a viscosity of 10,000 cPs. Owing to their dispersibility in water, low-molecular-weight polymers are correlated with higher rates of drug release (39). With a medium molecular weight, the plasticizer glycerol increases the solution viscosity, reduces brittleness, and fortifies the OF formulations. Furthermore, it facilitates robust intermolecular interactions between cellulose chains, thereby reducing the intermolecular tension along the entire polymer chain. Glycerol films have superior properties when compared to films manufactured using sorbitol or polyethylene glycol (PEG) (40). Glycerol is a hydrophilic substance that enhances the films flexibility, while reduces the strength when subjected to stress. Conversely, when the glycerol concentration increases, the elongation and moisture content also increase (40). Thus, the addition of a plasticizer is mandatory to improve the mechanical properties of the OFs.

**Figure 1:**
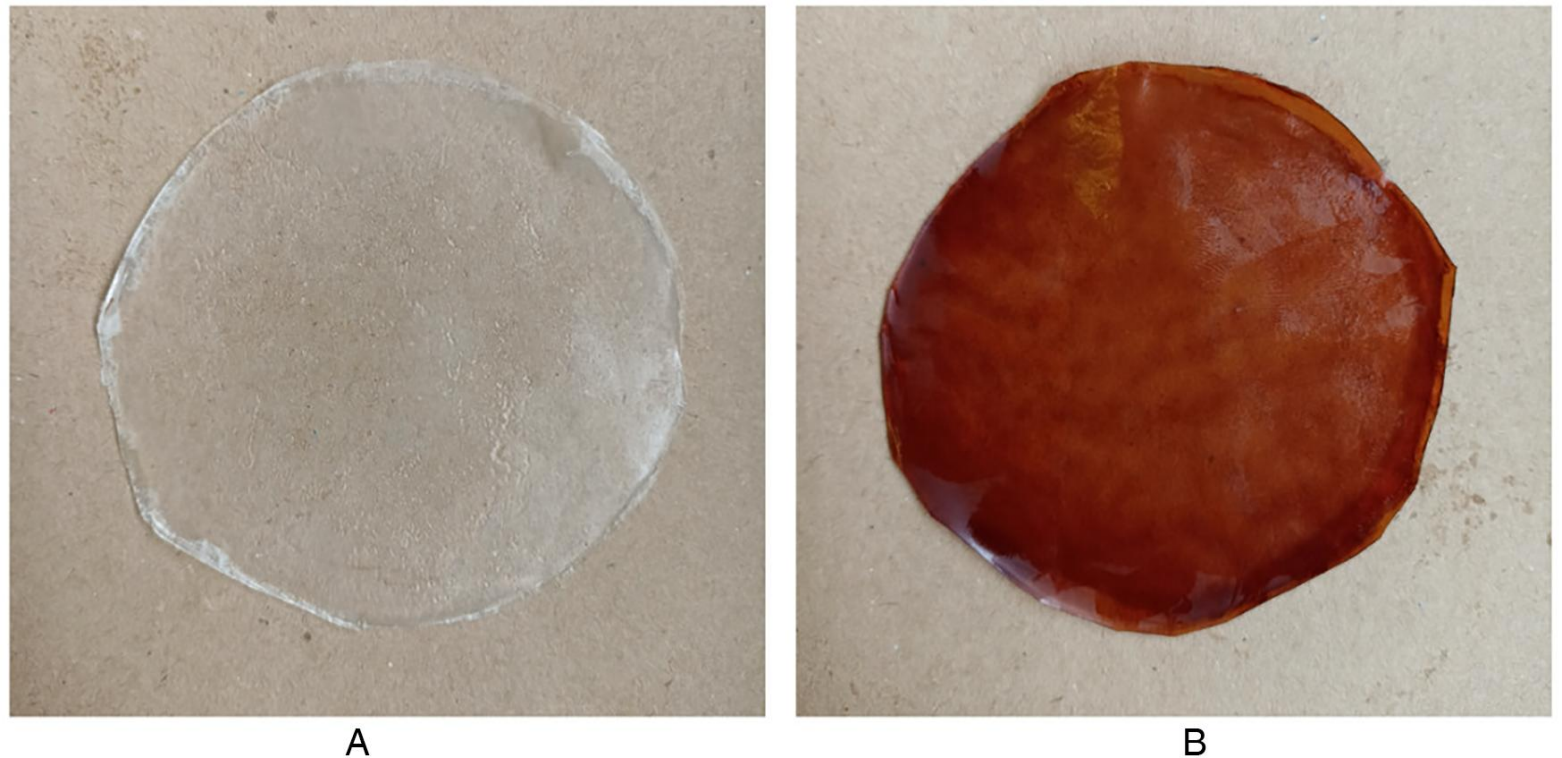
Formulated OFs after detachment from petri dishes: **(A)** placebo film; **(B)** CS-loaded oF.

The thin, transparent, homogeneous, flexible, and mucoadhesive coatings produced by the HPMC E10M formulation exhibited no visible fissures or air bubbles. CS-loaded OFs exhibited a brown coloration (Figure 1) attributed to the natural color of the extract, being not indicative of degradation. The quantitative transparency analysis, as recommended by previous studies (41, 42), confirmed that the films retained light transmittance values consistent with visually transparent materials. Although this mild discoloration could influence the patient perception, its intensity remained within acceptable limits for oral delivery systems and is unlikely to compromise therapeutic application.

### 3.2 Characterization of OFs

#### 3.2.1 Mechanical properties

The mechanical features of an optimal OF is determined by a variety of factors, including the microstructural network and constituents, interplay between matrix additives and preparation conditions, plasticizer, and pre-existing intermolecular forces (43–45). Table 1 summarizes the results obtained for the formulated OFs.

**Table 1:** Mechanical properties of formulated oral films (OFs), placebo and loaded with *C. sativa* shells (CS) extract (*n* = 3). Results are expressed as mean ± SD.

Formulation	Thickness (µm)	Rupture tensile strength (MPa)	Elongatio*n* (%)	Young's modulus (MPa)	Folding endurance
Placebo OFs	136 ± 8^a^	47.76 ± 1.11^a^	60.18 ± 7.43^a^	1,245.28 ± 26.43^a^	>300
CS-loaded OFs	125 ± 9^a^	43.05 ± 1.29^a^	75.28 ± 7.27^a^	1,141.74 ± 39.71^b^	>300

Different letters (a, b) in the same column means significant differences between samples (*p* < 0.05).

In addition to affecting mucoadhesion and oral comfort, film thickness is an essential factor related to the accuracy of the drug dosage. The barrier characteristics of the buccal mucosa and the rate of dissolution and disintegration may also be affected by thickness. Placebo and CS-loaded films (136 and 125 µm, respectively) showed no significant differences (*p* = 0.23). These values suggested that the formulations were relatively thin, which is advantageous since they may contribute to a pleasant sensation when applied to the buccal mucosa. As previously reported, the ideal thickness of oral thin films should be between 50 and 1,000 µm (26).

OFs must possess a substantial tensile strength to endure the stress associated with its production, packaging, transportation, and routine handling, because an insufficient tensile strength will result in rapid drug release from the matrix. As shown in Table 1, no statistical differences (*p* = 0.35) regarding tensile strength were observed between placebo and CS-loaded OFs (47.76 MPa and 43.05 MPa, respectively). Despite the slight decrease, the tensile strength of both film types exceeds the minimum thresholds required for orodispersible films to maintain integrity during handling and administration. According to Preis et al. (46), commercial orodispersible films typically exhibit tensile strength values ranging from 0.34 to 4.32 MPa, with a Young's modulus of up to 512 MPa, being still considered suitable for administration. The values observed in the present study (over 43 MPa) are an order of magnitude higher, demonstrating that the mechanical integrity and functionality of the CS-loaded films remain uncompromised. Therefore, the observed slight reduction in tensile strength does not impact the usability of the films and may even contribute to improve flexibility, which is desirable in this type of formulation.

Tedesco et al. studied the production of an OF with HPMC E15 incorporating peanut skin extract and reported a maximum tensile strength of 26.63 MPa (47). In another study, Borges et al. evaluated the tensile strength of marketed OFs and reported values between 1.47 and 33.91 MPa (48). Therefore, the tensile strength of the developed OFs was better than that reported in these studies.

Elongation is an indicator of flexibility, in which the concentrations of polymer, plasticizer, and extract are key aspects. CS-loaded OFs revealed a similar elongation percentage (75.28%) to placebo OFs (60.18%), with no significant differences (*p* = 0.42). According to Kola et al., the ethanolic extract from pomegranate seeds reduced the tensile strength of a cellulose film and increased the elongation at break (49). This effect could be attributed to the presence of (poly)phenols, indicating that the extract probably acted as a plasticizer, increasing the molecular attraction and forming new hydrogen bonds between the cellulose chains and extract (40, 49).

The tensile strength and elongation results corroborate the Young's modulus values, indicating resistance to deformation. As the tensile strength decreased and the elongation percentage increased, the Young's modulus decreased. The Young's modulus of the placebo OFs was significantly higher (1,245.28 MPa) than that of the CS-loaded OFs (1,141.74 MPa) (*p* = 0.04). Notably, the range values of both films align with those of commercial ones, which varied between 51.25 MPa and 1,824 MPa (50).

After being folded more than 300 times in the same place, the OFs were still unbroken, without signs of degradation, and showed a high level of flexibility, probably due to the presence of glycerol, denoting high mechanical strength. These results are in line with previous studies demonstrating the excellent mechanical performance of HPMC polymers (51).

#### 3.2.2 Physicochemical properties

The physicochemical properties of OFs, such as weight, moisture content, swelling capacity, disintegration time, and surface pH, are crucial for the patient compliance. As reported in Table 1, the placebo and CS-loaded OFs weighed 18 and 19 mg/cm^2^, respectively, without significant differences (*p* > 0.05). These results are not directly proportional to the thickness, as the CS-loaded OFs are heavier but thinner than the placebo ones. A possible explanation might be the higher density of the extract when compared to water.

OFs' mucoadhesion (related to comfort application) and stability (specifically microbial contamination) are highly influenced by the moisture content. A suitable water content not only mitigates the fragility of the OFs, but also acts as a powerful plasticizer (23). According to Nair et al. (20), the ideal moisture content of the OFs should be less than 5%. The results obtained for the placebo and CS-loaded OFs were approximately 4%, without significant differences (*p* > 0.05) (Table 2). It was not surprising that adding the CS extract to the polymeric matrix had no effect on the OFs humidity.

**Table 2:** Physicochemical properties of formulated orodispersible films (OFs), placebo and loaded with C. sativa shells (CS) extract (*n* = 3). Results are expressed as mean ± SD.

Formulation	Weight (mg/cm^2^)	Moisture content (%)	Swelling capacity (%)	Disintegration time (min:s)	Surface pH
Placebo OFs	18.0 ± 1.25^a^	4.36 ± 0.37^a^	9.13 ± 0.19^a^	18:35 ± 1:17^a^	6.98 ± 0.09^a^
CS-loaded OFs	19.0 ± 0.25^a^	4.25 ± 0.35^a^	7.31 ± 0.89^b^	20:43 ± 1:38^a^	6.94 ± 0.19^a^

Different letters (a, b) in the same column means significant differences between samples (*p* < 0.05).

The rate of drug release and mucoadhesive properties of the OFs, which are affected by the structure and composition of the polymeric matrix, can be determined through swelling evaluation (52). Upon application to the oral mucosa, water molecules permeate the OFs membrane, providing hydration to the polymer matrix. The increase in OFs volume induced by hydration facilitates the drug diffusion, in this study the CS extract. The placebo OFs exhibited a higher swelling capacity (9.13%) than the CS-loaded OFs (7.31%) owing to the hydrophilic nature of HPMC. HPMC is a hydrophilic polymer that rapidly absorbs water and swells, creating a gel-like network that supports mucoadhesion via hydrogen bonding with mucin (53, 54). When CS extract is incorporated, the polyphenols likely interact with the hydroxyl groups of HPMC, forming new intermolecular bonds that reduce the number of water-binding sites and creating a more compact matrix. This structural densification slows the water diffusion into the film, leading to reduced swelling and slower polymer relaxation. The adhesion that occurs when swelling begins leads to the formation of weak bonds. A hydration level increase corresponds to an increase in mucoadhesive strength. On the other hand, the strength rapidly decreases when the polymer becomes overhydrated and the interface becomes disentangled from the tissue (55).

The disintegration time indicates the onset of the drug action. A low disintegration time leads to a faster release and absorption of the loaded drug through the oral mucosa. The disintegration time was evaluated in artificial saliva (pH 6.8) at 37°C to mimic the oral conditions. Placebo OFs disintegrated after 18 min and CS-loaded OFs after more than 20 min, without significant differences (*p* > 0.05). These values correlate with the swelling capacity of the OFs, as a higher swelling capacity leads to a faster disintegration (48).

Surface pH is an important attribute of OFs intended to be applied to mucous membranes, since it can be related to eventual local damage or irritation, causing discomfort to patients. The pH of human saliva ranges from 6.2 and 7.6, with a specific value of 6.3 for the buccal mucosa (56). As attested, the OFs presented a pH between 6.94 and 6.98 and were considered suitable for oral application, without risk of irritation or inflammation of the buccal mucosa.

#### 3.2.3 Stability

Stability assays are essential for the development of new drug delivery systems. Additionally, these tests should be performed on OFs to determine the behavior of their components and identify any potential degradations or interactions. Products must be stable after manufacturing to comply with the standards set by the International Council of Harmonization (ICH), with packaging providing mechanical protection and acting as a crucial barrier against light, moisture, and oxygen (26, 57). Therefore, CS-loaded OFs were evaluated for short-term and accelerated stability studies (Table 3).

**Table 3:** Effects of storage at room temperature and accelerated conditions on the properties of the C. sativa shells (CS) extract loaded Oral Films (OFs) (*n* = 3). Results are expressed as mean ± SD.

CS-loaded OFs						
Storage conditions (25°C/65% RH)						
Time (days)	Rupture tensile strength (MPa)	Elongation (%)	Folding endurance	Disintegration time (min:s)	Surface pH	Physical appearance
0	43.05 ± 1.29	75.28 ± 7.27	>300	20:43 ± 01:38	6.94 ± 0.19	–
15	42.78 ± 1.45	76.31 ± 5.79	>300	19:33 ± 02:55	6.89 ± 0.24	No change
30	43.20 ± 1.34	77.02 ± 5.54	>300	20:13 ± 01:36	6.91 ± 0.13	No change
60	44.05 ± 2.03	77.89 ± 3.41	>300	21:01 ± 00:59	6.92 ± 0.09	No change
90	41.05 ± 1.79	76.12 ± 4.76	>300	20:22 ± 01:41	6.95 ± 0.21	No change
Accelerated conditions (40°C/75% RH)						
0	41.27 ± 1.35	76.82 ± 5.86	>300	19:27 ± 01:13	6.92 ± 0.15	-
15	42.35 ± 2.64	75.87 ± 6.15	>300	20:14 ± 02:41	6.87 ± 0.18	No change
30	41.63 ± 1.67	74.23 ± 4.69	>300	21:53 ± 02:05	6.94 ± 0.26	No change
60	40.97 ± 2.53	74.75 ± 2.71	>300	22:05 ± 01:52	6.83 ± 0.21	No change
90	39.05 ± 1.39	73.87 ± 3.67	>300	22:43 ± 00:43	6.74 ± 0.18	No change

As can be observed, the folding endurance of the OFs remained in the acceptable range of more than 300-fold even after 90 days of storage at 40°C/75% RH. Similarly, no significant differences were observed in terms of tensile strength, elongation, physical appearance, disintegration time, or surface pH. The absence of significant differences for all parameters at room temperature (25°C/65% RH) or under accelerated conditions (40°C/ 75% RH) indicate a good physicochemical stability for the developed OFs. To the best of our knowledge, this is the first study that assessed the stability of OFs using natural extracts as active ingredients.

#### 3.2.4 Phenolic content and antioxidant/antiradical activities

One of the main triggers in the development of OM is oxidative stress, which can be minimized with the use of natural antioxidants, particularly delivery systems. Table 4 summarizes the TPC and antioxidant/antiradical activities of the OFs.

**Table 4:** Total phenolic content (TPC) and antioxidant activity (FRAP and DPPH) of formulated oral films (OFs), placebo and loaded with C. sativa shells (CS) extract (*n* = 3). Results are expressed as mean ± SD.

Formulation	TPC (mg GAE/g film)	DPPH (mg TE/g film)	FRAP (µmol FSE/g film)
Placebo OFs	ND	ND	ND
CS-loaded OFs	37.05 ± 1.20	143.42 ± 3.41	0.142 ± 0.01

ND, Not Determined.

As expected, placebo OFs did not show results for these assays. In a previous study, our team evaluated the phenolic content of the dry CS extract and reported a TPC value of 239.53 mg GAE/g DW (14). Considering that the extract was used as the solvent at 25% (*v/v*) for the CS-loaded OFs, the obtained TPC values closely aligned with this result (59.88 mg GAE/g of film) (14).

The same ratio was not observed in the antioxidant/antiradical assays. However, due to the potential interference of the polymer or plasticizer in these assays, it is expected that the OFs would yield lower results than the dry extracts. The CS-loaded OFs achieved a result of 143.43 mg TE/g film (dry extract: 426.88 mg TE/g DW) and 0.142 µmol FSE/g film (dry extract: 4,092.98 µmol FSE/g DW) for the DPPH and the FRAP assays, respectively. A potential reason may be the low ability of the natural antioxidants present in the extract to scavenge DPPH free radicals or to bind to the FRAP ions, as they are entrapped in the polymeric matrix, particularly when the polymer is selected for its sustained release profile (58).

Nonetheless, it should be highlighted that natural polyphenols are susceptible to oxidation and degradation under thermal or environmental stress, which can occur during film drying or prolonged storage.

#### 3.2.5 SEM

**Figure 2:**
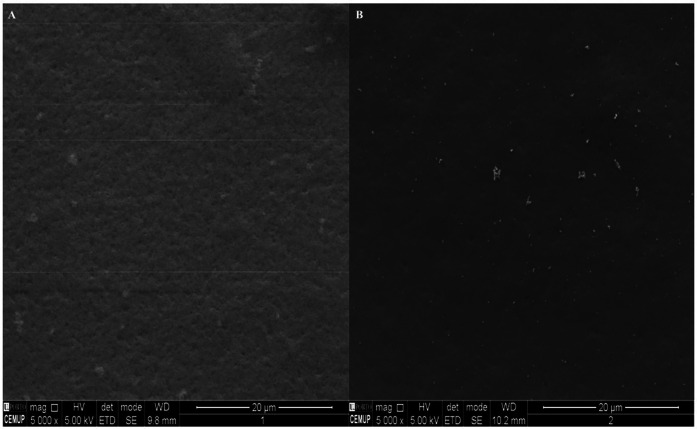
Scanning electron microscopy (SEM) micrographs of the produced oral films: **(A)** placebo film; **(B)** CS-loaded OF (5,000 x).

For ideal buccal application, the morphology of OFs should be homogeneous to ensure a uniform distribution of the bioactive content through the polymeric mixture. Additionally, interactions between drugs, polymers, and plasticizers may result in a rough OFs surface (26). Figure 2 represent the OFs surfaces observed by SEM.

The microscopic appearance of the OFs placebo (A) presented a homogeneous, continuous, and smooth surface, despite the presence of white dots in both OFs that were more noticeable in the CS-loaded OFs (B). These results are in line with the ones of Porfírio et al.*,* who also observed small white dots in HPMC films, while adding zidovudine and lamivudine, suggesting the precipitation of the polymer involved (59).

#### 3.2.6 Polymer and extract interactions

**Figure 3:**
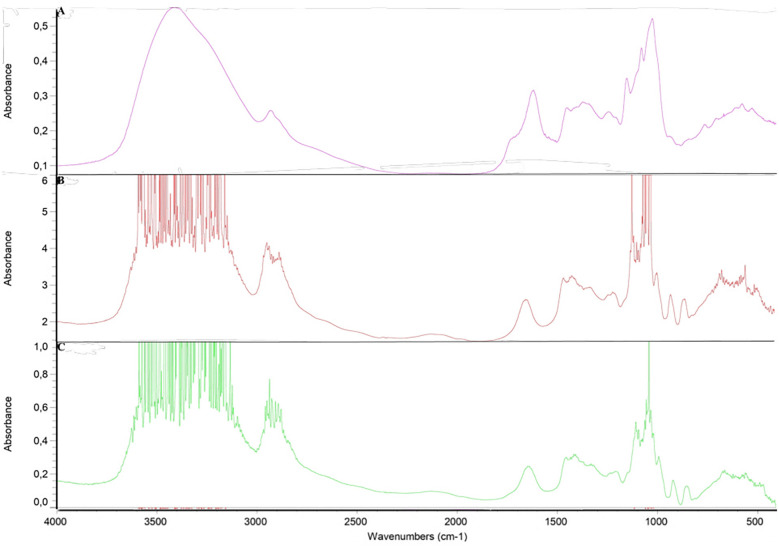
FTIR spectrum: **(A)** *C. sativa* shells (CS) extract; **(B)** placebo film; **(C)** CS-loaded OF.

FTIR was employed to assess the impact of CS extract incorporation on the intermolecular forces within the HPMC E10M OFs (Figure 3).

The extract introduction resulted in the emergence of new peaks in the OFs, suggesting potential modifications to the chemical structure of the HPMC matrix. Notable differences were observed in the 3,600–3,100 cm^−1^ region, associated with the stretching vibrations of the O-H bonds (hydroxyl groups) and N-H bonds (amines and amides) (60, 61). These modifications may signify a change in the hydrogen-bonding patterns or the introduction of new functional groups containing O-H or N-H bonds. Furthermore, the presence of hydroxyl groups in compounds such as catechin and epicatechin may contribute to modifications in this region (62).

The alterations observed in the 2,950–2,850 cm^−1^ region, mainly linked to the stretching vibrations of C-H, may result from the presence of alkaloids, flavonoids, phenolic acids, and stilbenes. The 1,150–1,000 cm^−1^ region, which is characteristic of the HPMC and is associated with the C-O stretching, displayed differences, suggesting modifications in the cellulose backbone or the introduction of functional groups from the extract (63). Compounds such as catechin and rutin, with distinct C-O stretching characteristics, contribute to these modifications (62, 64).

Based on the chemical nature of the constituents, it is not expect drastic shifts or new peak formations, supporting the physical compatibility of the components.

#### 3.2.7 DSC

**Figure 4:**
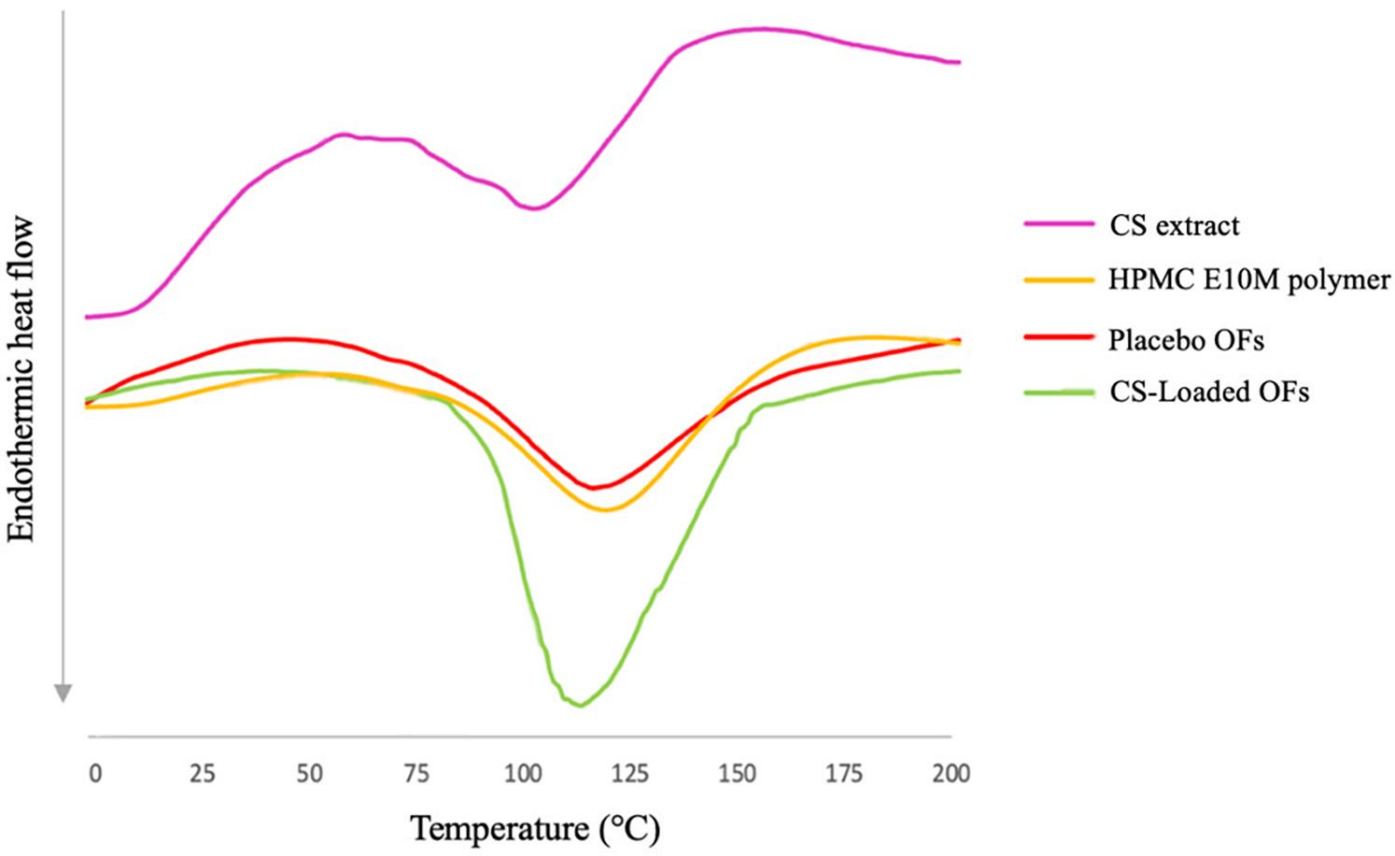
DSC thermograms of *C. sativa* shells (CS) extract, pure HPMC polymer and produced oral films (placebo and CS-loaded OF).

The DSC thermograms, represented in Figure 4, showed a broad endothermic peak for the lyophilized CS extract at 103.76°C, which may correspond to its melting point.

Since there is a mixture of (poly)phenols in the matrix that can interact with each other, the thermal degradation may occur with the conversion of some compounds into different phenolic acids (14). However, to the best of our knowledge, there is a lack of information regarding DSC analyses of CS extract. The thermograms of the pure HPMC E10M polymer and the placebo films exhibit shallow, broad endothermic peaks at 119.54°C, and 115.32°C, respectively. Conversely, the CS-loaded OFs displayed a more pronounced peak at 118.21°C, indicating a greater degree of heat fusion. These results suggest that the inclusion of (poly)phenols requires a greater amount of energy to disrupt the interactions between (poly)phenols and the film matrix (65). Based on the thermograms obtained for the mixture, no significant interactions were observed between the extract and the excipients, being HPMC E10M and glycerin compatible with the CS extract.

#### 3.2.8 Ex vivo mucoadhesive strength

Mucoadhesion is influenced by the polymers physicochemical characteristics, such as charge, concentration, functional groups, and environmental factors. In fact, low-molecular-weight polymers are more effective at permeating mucous, whereas high-molecular-weight polymers become entangled with mucin more frequently. HPMC and other polymers in the cellulose derivative class have hydroxyl and carboxyl groups substituted for their natural cellulose backbones. Carboxyl groups attract water, resulting in significant diffusion and enlargement of polymer chains. Pharmacokinetics are improved through ionic and hydrogen bonding with mucin oligosaccharides, which prolongs the residence time of carboxylic groups at the application site. Furthermore, the flexibility of the polymeric matrix facilitates the formation of a greater number of hydrogen bonds (43).

The mucoadhesive strength of the OFs was evaluated in porcine buccal mucosa using a texture analyzer, and the detachment force and work of adhesion were measured (Table 5).

**Table 5:** Mucoadhesive strength of formulated orodispersible films (OFs), placebo and loaded with C. sativa shells (CS) extract (*n* = 3).

Formulation	Detachment force (N)	Work of adhesion (N/mm)
Placebo OFs	1.89 ± 0.41	0.26 ± 0.01
CS-loaded OFs	1.77 ± 0.23	0.19 ± 0.02

The detachment force was much lower than the one reported in other studies (66, 67), and no significant differences were detected between the CS-loaded OFs and the placebo ones (*p* > 0.05). For example, Al-Dhubiab et al., using polymer blends of HPMC, Eudragit and Carbopol, reported detachment forces of 7.89–8.34 N for different polymer ratios (66). Mady et al. obtained detachment force values between 0.306 and 0.416 N for a polymeric mixture composed of carboxymethyl cellulose and polyvinylpyrrolidone with propylene glycol and Tween (67). It is likely that the hydrophilicity associated with the thickness of the HPMC OFs leads to rapid disintegration, which reduces the mucoadhesive characteristics. The high solubility of polymers in water can be a positive aspect since, after hydration, the OFs start to disintegrate due to dissolution.

### 3.3 Cytotoxicity

**Figure 5:**
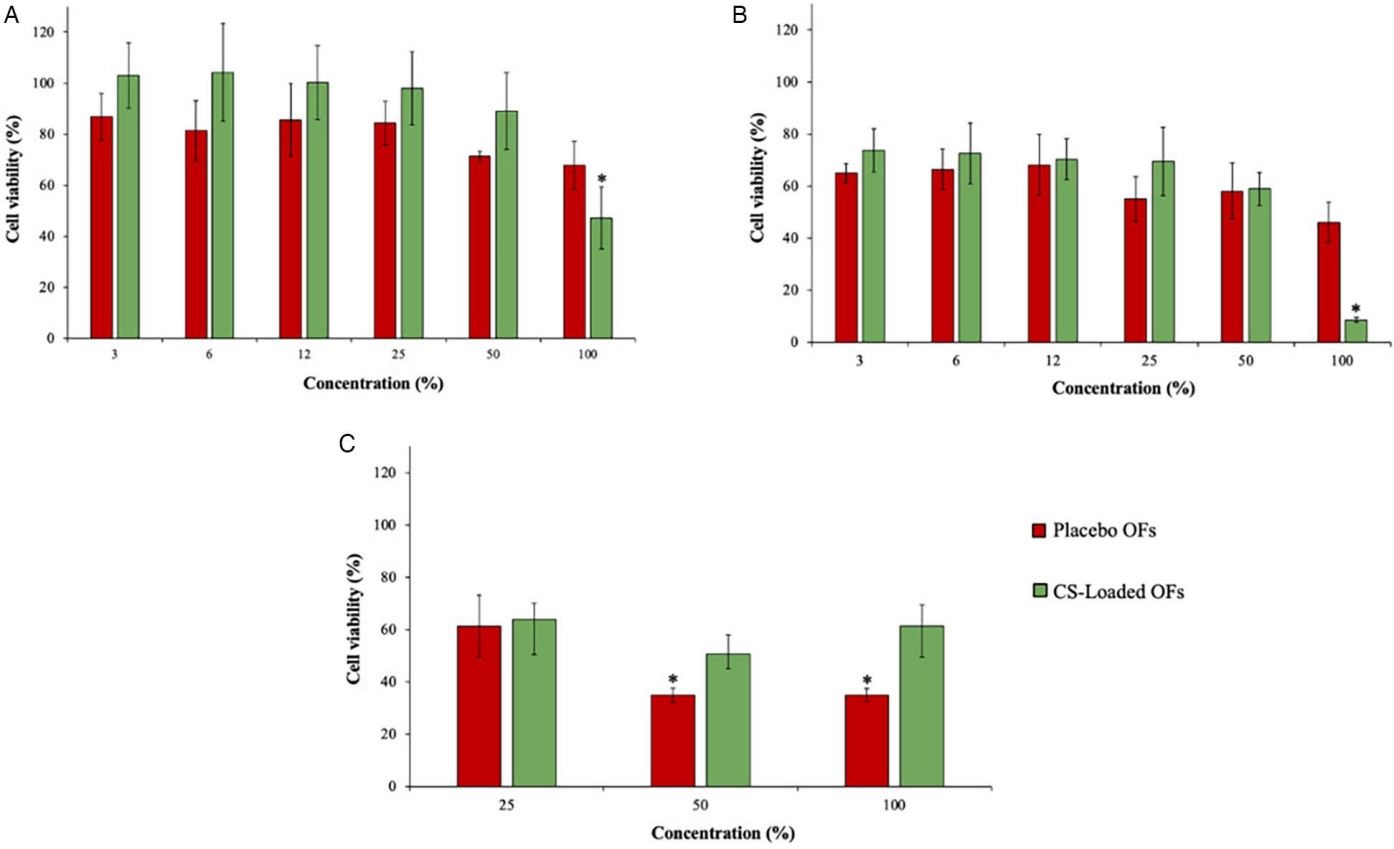
Effects of placebo and CS-loaded OFs exposure on the viability of TR146 **(A)**, HSC-3 **(B)** and HaCaT **(C)** cell lines at different concentrations, measured by the MTT assay (*n* = 3). * means significant differences between the different tested concentrations (*p* < 0.05).

In addition to the mechanical and biodegradable properties, polymer biocompatibility is a critical factor for its use in pharmaceutical applications. The MTT assay was performed on TR146, HSC-3, and HaCaT cells after 24 h of exposure to the OFs, as illustrated in Figure 5.

TR146 and HSC-3 are human tumorigenic oral cell lines, commonly used as cellular models for studies on the oral cavity, while HaCaT is a human immortalized keratinocyte line derived from normal skin that has similarities with epidermis and epithelial tissue of the oral mucosa (68, 69). The following criteria were used to classify cellular viability: viability > 100%, indicating no toxicity (class 0); viability = 0%, revealing the highest toxicity (class 5); and 75%–99%, 50%–74%, 24%–49%, and 1%–25% viability were categorized as classes 1, 2, 3, and 4, respectively (70).

The toxicity of CS-loaded OFs was higher than the placebo at the highest tested concentration (100%, no dilution), with viabilities of 47.22% and 67.91%, respectively. Similarly, as the placebo OFs led to a viability of 46.00% in the HSC-3 cell line, exposure to CS-loaded OFs resulted in a viability of 8.62%. This evidence confirmed the cytotoxic effects of the CS extract on both tumorigenic cell lines at the highest tested concentration. However, for diluted concentrations of OFs, this effect was not observed. Regarding HaCaT cells, a higher viability was observed after exposure to the highest concentration of the CS-loaded OFs (61.2%) than after exposure to the placebo OFs (35.06%), being categorized as classes 2 and 3, respectively. These results confirm the antitumor effect of the CS extract owing to the selectivity of its cytotoxic effects. Moreover, the reduced cellular viability may be due to the high viscosity of the polymer, which prevents the proper cellular division in 2D cellular models.

### 3.4 Metabolomic profile

#### 3.4.1 *in vitro* permeability

The permeation of natural compounds from prepared OFs must be evaluated to assess their absorption across the buccal epithelium. In this study, the safety and bioavailability of the developed OFs were evaluated in an *in vitro* buccal model composed by TR146 cells. Tables 6, 7 summarize the permeated compounds identified and quantified after the *in vitro* permeation assay of the CS extract and the CS-loaded OFs at different time points (for up to 240 min). Supplementary Table S1 presents the putative annotations of the compounds in the CS extract and the CS-loaded OFs. Chestnut shells are known to contain polymerised phenolic compounds, including procyanidins and proanthocyanidins, which can present molecular weights exceeding the *m/z* 600 limit used in our LC-ESI-LTQ-Orbitrap-HRMS acquisition. In the present work, the acquisition range was set to *m/z* 100–600 to prioritize the identification and quantification of low- to medium-molecular-weight phenolics and related metabolites that are more likely to permeate through the buccal mucosa due to their smaller size. Our focus was on compounds with potential for buccal absorption, with lower molecular weights. As expected, the *in vitro* and *ex vivo* assays revealed no bioactive compound permeation in the placebo films.

**Table 6:** Permeation of *C. sativa* shells extract compounds through the *in vitro* buccal model (TR146 cells) at different time points (*n* = 3). Results are expressed as mean ± SD.

Class	Subclass	Compound/Metabolite	Permeation (%)							
			Time (min)							
			15	30	45	60	90	120	180	240
Benzene	Benzoic acids	Methyl gallate	37.24 ± 0.04^a^	ND	ND	ND	ND	ND	ND	ND
Carboxylic acids	Amino acids, peptides	Pyroglutamic acid	13.92 ± 0.12^a^	15.39 ± 1.05^a^	18.22 ± 1.41^b^	23.34 ± 0.88^b^	22.87 ± 1.52^b^	23.68 ± 1.84^b^	21.35 ± 0.97^b^	28.47 ± 2.52^b^
	Dicarboxylic acids	Succinic acid	20.51 ± 1.62^b^	14.86 ± 2.71^a^	16.09 ± 1.85^a^	14.96 ± 0.46^a^	19.54 ± 0.94^b^	20.71 ± 1.67^b^	21.62 ± 0.61^b^	23.76 ± 1.68^b^
	Tricarboxylic acid	Isocitric acid	0.04 ± 0.01^a^	0.12 ± 0.03^a^	0.15 ± 0.01^a^	0.15 ± 0.01^a^	0.24 ± 0.03^a^	0.33 ± 0.02^a^^,^^b^	0.54 ± 0.04^a^^,^^b^	0.70 ± 0.01^b^
Dibenzylbutane lignans	Dibenzylbutanediol lignans	Secoisolariciresinol	ND	ND	ND	ND	ND	ND	22.32 ± 0.12^a^	ND
Fatty Acyls	Fatty acids	Hydroxyadipic acid	25.51 ± 1.51^a^	54.36 ± 0.58^b^	24.25 ± 0.84^a^	24.30 ± 0.10^a^	25.06 ± 0.79^a^	34.93 ± 2.05^a^	29.87 ± 1.69^a^	62.63 ± 2.33^b^
		Sativic acid	1.85 ± 0.01^a^	3.02 ± 0.05^a^	3.77 ± 0.02^a^	4.46 ± 0.03^a^	6.17 ± 0.08^a^^,^^b^	7.52 ± 1.13^a^^,^^b^	10.80 ± 0.16^a^^,^^b^	12.34 ± 0.09^b^
		Sebacic acid	121.43 ± 3.045^a^	108.30 ± 1.07^a^	117.63 ± 2.87^a^	122.27 ± 3.63^a^	120.02 ± 4.05^a^	113.74 ± 2.24^a^	117.88 ± 1.74^a^	101.09 ± 3.73^a^
Flavonoids	Flavans	Epicatechin	97.57 ± 1.07^a^	96.86 ± 2.25^a^	97.35 ± 1.74^a^	98.05 ± 3.81^a^	97.75 ± 5.73^a^	97.73 ± 6.01^a^	97.36 ± 2.57^a^	96.86 ± 1.86^a^
	Flavonoid glycosides	Quercetin 3-*O*-galactoside	ND	ND	ND	49.94 ± 0.98^a^	ND	ND	ND	ND
	Flavones	Isorhamnetin	99.26 ± 2.34^a^	99.73 ± 1.83^a^	98.36 ± 3.74^a^	99.10 ± 2.96^a^	99.00 ± 4.70^a^	98.36 ± 1.07^a^	99.08 ± 2.69^a^	98.36 ± 1.91^a^
Organooxygen	Alcohols and polyols	Caffeoyl quinic acid	ND	ND	29.74 ± 0.73^a^	ND	ND	60.35 ± 1.67^b^	29.76 ± 0.92^a^	30.86 ± 0.63^a^
		Myo-Inositol	39.51 ± 0.87^a^^,^^b^	79.23 ± 1.43c	12.98 ± 0.01^a^	33.80 ± 0.08^a^^,^^b^	30.46 ± 0.05^a^^,^^b^	41.34 ± 1.01^a^^,^^b^	25.02 ± 0.09^a^^,^^b^	56.50 ± 0.37^c^
		Neochlorogenic acid	ND	ND	ND	ND	ND	ND	14.58 ± 0.08^a^	ND
	Carbohydrates	Verbascose	3.25 ± 0.01^a^	4.31 ± 0.08^a^	5.11 ± 0.02^a^	5.46 ± 0.01^a^	8.41 ± 0.03^a^	10.23 ± 0.11^b^	13.92 ± 0.06^b^	18.05 ± 0.03^b^
Phenolic acids	Hydroxybenzoic acids	3-Hydroxybenzoic acid	13.57 ± 0.05^a^	13.06 ± 0.03^a^	14.09 ± 0.01^a^	17.29 ± 0.01^a^	16.17 ± 0.06^a^	29.12 ± 0.19^b^	16.86 ± 0.23^a^	24.23 ± 0.45^b^
		Protocatechuic acid	39.32 ± 0.96^b^	43.86 ± 1.62^b^	42.03 ± 0.79^b^	38.29 ± 0.86^b^	37.52 ± 0.97^b^	25.84 ± 1.03^a^	40.40 ± 0.82^c^	32.88 ± 0.87^b^
Phenols	Benzenoids	Pyrocatechol	34.44 ± 0.73^b^	33.04 ± 0.46^b^	16.02 ± 0.81^a^	ND	15.89 ± 0.05^a^	ND	16.11 ± 0.01^a^	20.56 ± 0.14^a^^,^^b^
	Methoxyphenols	Homovanillic acid	93.51 ± 2.52^b^	92.34 ± 1.55^b^	92.13 ± 1.08^b^	93.10 ± 0.94^b^	94.47 ± 3.00^b^	90.93 ± 2.74^b^	91.20 ± 1.73^b^	87.20 ± 2.06^a^
		Vanillin	8.10 ± 0.02^a^	23.12 ± 0.56^b^	28.35 ± 0.83^b^	28.43 ± 0.41^b^	42.18 ± 1.08^c^	27.71 ± 0.72^a^	53.91 ± 1.07^d^	52.90 ± 0.73^d^

Different letters (^a^^,b^^,c^^,d^) in the same line means significant differences (*p* < 0.05).

ND, not determined.

**Table 7:** Permeation of compounds present in formulated oral films with C. sativa shells extract incorporated through the *in vitro* buccal model (TR146 cells) at different time points (*n* = 3).

Class	Subclass	Compound/Metabolite	Permeation (%)							
			Time (min)							
			15	30	45	60	90	120	180	240
Carboxylic acids	Amino acids, peptides	Pyroglutamic acid	17.03 ± 0.23^a^	39.63 ± 0.24^a^^,^^b^	43.32 ± 1.12^a^^,^^b^	46.32 ± 2.65^a^^,^^b^	65.40 ± 0.82^b^	66.95 ± 3.71^b^	103.24 ± 2.71^c^	111.34 ± 1.62^c^
	Dicarboxylic acids	Succinic acid	44.20 ± 2.19^a^	64.44 ± 1.03^c^	56.70 ± 1.23^b^	57.45 ± 0.78^b^	79.12 ± 4.41^c^	88.23 ± 1.26^c^	103.25 ± 1.42^d^	100.86 ± 1.75^d^
	Tricarboxylic acid	Isocitric acid	0.15 ± 0.001^a^	0.29 ± 0.05^a^	0.50 ± 0.06^a^	0.62 ± 0.02^a^	1.04 ± 0.01^b^	1.40 ± 0.04^b^	2.77 ± 0.01^b^	3.38 ± 0.06^b^
Fatty Acyls	Fatty acids	Sativic acid	7.64 ± 0.45^a^	13.72 ± 0.58^b^	19.16 ± 0.87^b^	21.17 ± 2.41^b^	34.01 ± 1.38^c^	43.66 ± 3.51^c^	58.08 ± 0.63^d^	71.88 ± 1.47^d^
Flavonoids	Flavones	Isorhamnetin	105.62 ± 3.71^a^	105.51 ± 3.25^a^	105.05 ± 2.24^a^	101.34 ± 2.95^a^	98.29 ± 2.14^a^	99.38 ± 1.47^a^	98.29 ± 1.94^a^	100.20 ± 3.56^a^
Organooxygen	Alcohols and polyols	Caffeoylquinic acid	ND	ND	47.99 ± 1.41	ND	ND	95.85 ± 2.72	39.15 ± 0.84	69.64 ± 2.61
	Carbohydrates	Verbascose	2.14 ± 0.01^a^	4.25 ± 0.15^a^	4.45 ± 0.98^a^	4.86 ± 0.41^a^	7.90 ± 0.51^b^	11.25 ± 0.13^b^	16.17 ± 0.44^a^^,^^b^	20.82 ± 0.97^a^^,^^b^
Phenolic acids	Hydroxybenzoic acids	Protocatechuic acid	89.42 ± 3.08^b^	ND	85.88 ± 2.01^b^	94.08 ± 3.56^b^	96.88 ± 1.43^b^	106.06 ± 0.78^b^	84.76 ± 2.51^b^	75.80 ± 0.91^a^
Phenols	Benzenoids	Pyrocatechol	99.63 ± 2.51^c^	107.69 ± 3.06^c^	54.19 ± 1.35^a^	ND	57.35 ± 0.85^a^	ND	58.10 ± 1.73^a^	68.19 ± 2.65^b^
	Methoxyphenols	Homovanillic acid	106.01 ± 1.46^a^	104.89 ± 1.08^a^	105.41 ± 0.99^a^	103.62 ± 2.72^a^	105.25 ± 2.83^a^	109.09 ± 1.97^a^	106.07 ± 2.67^a^	110.05 ± 3.61^a^

Different letters (^a^^,b^^,c^^,d^) in the same line means significant differences (*p* < 0.05).

ND, not determined.

Regarding the CS extract, a total of 20 compounds were presumptively annotated in the *in vitro* buccal model permeation, with a higher abundance of (poly)phenols (7 compounds, representing 35%) and organic acids (5 compounds, representing 25%), followed by lipids (3 compounds, representing 15%), alcohols or polyols (3 compounds, representing 15%), carbohydrates (1 compound, representing 5%), and amino acids and derivatives (1 compound, representing 5%) (Supplementary Table S2). From the CS extract, 16 compounds permeated through the *in vitro* buccal model after up to 4 h of assay. Sebacic acid exhibited the highest permeation at most timepoints, reaching a permeability of 101.09% after 240 min, followed by isorhamnetin (98.36%), epicatechin (96.86%), and homovanillic acid (87.20%). Reactive oxygen species (ROS) play a central role in OM pathogenesis, since epithelial cell damage triggers apoptosis and upregulates several transcription factors (14). This cascade culminates in the induction of a pro-inflammatory state characterized by the production of cytokines, including tumor necrosis factor-α (TNF-α), interleukin-β and -6 (IL-β and IL-6) (14). Particularly, an increase in salivary IL-6 levels during the third week of treatment in patients with head and neck cancer is associated with the development of severe OM (71). Sebacic acid, a fatty acid present in royal jelly, has been associated with anti-inflammatory properties by reducing the expression of IL-6 induced by lipopolysaccharide (LPS) in human macrophages (72). Moreover, isorhamnetin has shown protective effects against H_2_O_2_^−^induced endothelial cell injury via antioxidative, anti-inflammatory, and anti-mitochondria-dependent apoptosis (73). Shin et al. demonstrated that epicatechin protected HaCaT cells from radiation-induced damage *in vitro* by inhibiting the ROS generation, preserving mitochondrial integrity, and suppressing MAPKs activation. Also, in a rat model, epicatechin improved the wound healing after radiation exposure in the oral cavity (74), while homovanillic acid had potent antioxidant properties in rats after olive oil consumption (75).

Regarding the CS-loaded OF-permeated samples, the results demonstrated a lower abundance of annotated compounds, probably due to the concentration of the CS extract used in the OFs (25%, *v/v*) as well as the evaporation process that occurs during the OFs production. Zelbiene et al. tested different types of polyacrylic acid gels with horse chestnut seed extract and demonstrated that the permeability of (poly)phenolic compounds was dependent on the polymer used, highlighting the importance of polymeric matrices for release purposes. This problem can be solved by incorporating a permeation enhancer (76). The concentration, structure, molecular size, hydrophilicity, and permeation time may also affect the compounds permeation through the oral mucosa (26, 77). Nonetheless, it should be highlighted that this cellular model has drawbacks, including the influence of oncogenesis on the permeability barrier (78). Pyroglutamic acid (PGA) achieved the highest permeation in CS-loaded OFs, reaching 111.34% after 240 min, followed by homovanillic acid (110.05%), succinic acid (100.86%), isorhamnetin (100.20%), protocatechuic acid (75.80%), and caffeoylquinic acid (69.64%). PGA is a low molecular weight carboxylic acid well known for its excellent hygroscopic properties, as well as its demonstrated antitumor and antimicrobial activities (79). Additionally, PGA has been associated with a decrease in the concentration of inflammatory cytokines within the *stratum corneum*, suggesting potential anti-inflammatory effects. Moreover, the synergy between PGA and antioxidants may enhance the restoration of physiological skin function. Therefore, CS-loaded OFs may represent a promising strategy for mitigating OM ulceration.

Protocatechuic acid (PCA) and caffeoylquinic acid are bioactive compounds with diverse health benefits due to their anti-inflammatory, antioxidant, antitumor, and antibacterial activities (80, 81). Moreover, *in vitro* and *in vivo* studies have attested the PCA's antiulcer activity and analgesic effects (80). During OM, the skin epithelial barrier is compromised, exposing patients to potential pathogenic bacteria, such as *Staphylococcus aureus* and *Escherichia coli*, increasing the risk of sepsis. Caffeoylquinic acid has demonstrated inhibitory effects against these bacteria, suggesting its capacity to mitigate secondary infections (81). It should be noted that these compounds showed higher permeations in the CS-loaded OFs than in the CS extract, indicating that the formulated delivery system is a good vehicle for these natural compounds.

**Figure 6:**
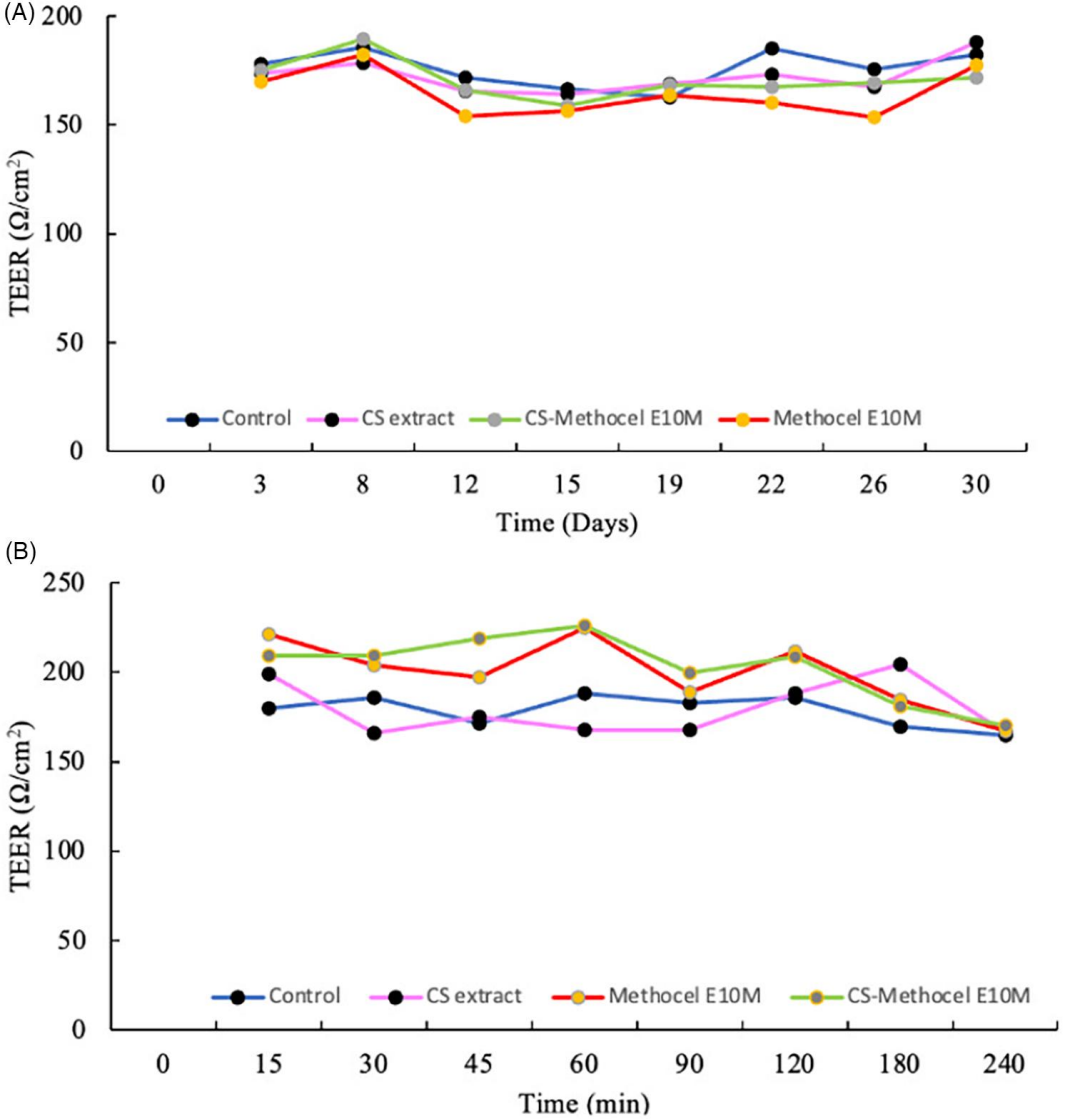
Transepithelial electrical resistance (TEER) measurements of the 3D buccal model (TR146 cells) monitored during: **(A)** 31 days and **(B)** 240 min of the permeability assay.

During the *in vitro* model assays, TEER is a decisive parameter for evaluating the integrity of the cell layers. In the present study, TEER was monitored for 31 days. As shown in Figure 6, the values increased on the 8th day, indicating that the TR146 cells grew and remained stable (165 ± 20 Ω/cm^2^). During the permeability assay, the values ranged between 166.0 and 210.0 Ω/cm^2^, demonstrating the viability of the cell culture. Mazzinelli et al. reported that TEER values between 150 and 200 Ω/cm^2^ are greater than those of healthy epithelium due to their derivation from carcinoma (82). However, these values were lower than the ones obtained for models with Caco-2 cells due to the absence of tight junctions (82). Therefore, the TR146 cell layers maintained the cellular morphology and integrity of the monolayer during the experiments.

#### 3.4.2 Ex vivo permeation

*Ex vivo* studies are used as screening permeation tools and offer the advantage of reducing labor and experimental costs when compared to *in vivo* animal studies. Porcine buccal mucosa is the standard animal model for oral permeability studies due to its close resemblance to the human buccal mucosa, as it is a non-keratinized tissue and has a similar enzymatic composition (83). In fact, the oral mucosa shows intermediate permeability properties between the epidermis and the gut, acting as a barrier to compound permeation (82). It is also worth noting that in buccal administration, the mucosal barrier properties rely on the structural and physicochemical features of the oral tissue and active ingredients (82). Therefore, an *ex vivo* permeation study was performed using a vertical Franz-type diffusion cell apparatus coupled to porcine buccal mucosa. Table 8 outlines the permeated compounds identified and quantified after the *ex vivo* permeation assay of the CS extract and CS-loaded OFs at different time points, up to 30 min under conditions that mimic the human oral environment, once the OFs disintegrate within 20 min.

**Table 8:** Permeation of compounds present in *C. sativa* shells extract (CS) and CS-loaded Orodispersible Films (OFs) in porcine buccal mucosa (Franz cells) up to 30 min (*n* = 3).

Class	Subclass	Compound/metabolite	Permeation (%)			
			CS extract		CS-loaded OFs	
			Time (min)			
			15	30	15	30
Carboxylic acids	Amino acids, peptides	L-Phenylalanine	8.67 ± 0.03^a^	8.57 ± 1.47^a^	ND	ND
		L-Tyrosine	7.39 ± 0.79^a^	7.26 ± 0.06^a^	74.20 ± 1.98^b^	76.52 ± 2.73^b^
		Acetyl-L-leucine	63.81 ± 2.85^a^	66.76 ± 0.28^a^	104.42 ± 3.78^b^	135.84 ± 5.25^b^
	Dicarboxylic acids	Fumaric acid	15.02 ± 0.04^a^	16.78 ± 0.19^a^	ND	ND
		Succinic acid	60.67 ± 0.21^b^	70.72 ± 3.61^b^	14.00 ± 0.63^a^	16.11 ± 0.72^a^
	Indoyl carboxylic acids	L-Tryptophan	37.21 ± 0.01^a^	27.24 ± 0.23^a^	ND	ND
	Tricarboxylic acid	Isocitric acid	15.13 ± 0.29^a^	13.91 ± 1.51^a^	19.05 ± 1.12^a^	19.02 ± 0.90^a^
Cinnamic acids	Hydroxycinnamic acids	Hydroxycinnamic acid Isomer II	67.14 ± 2.35^a^	64.79 ± 2.04^a^	ND	ND
Fatty Acyls	Fatty acids	Adipic acid	11.41 ± 0.03^a^	9.47 ± 0.07^a^	98.93 ± 2.05^b^	97.59 ± 2.36^b^
		Mevalonic acid	1.26 ± 0.01^a^	1.35 ± 0.01^a^	ND	ND
		Pimelic acid	65.10 ± 1.87^a^	67.06 ± 2.58^a^	76.61 ± 4.85^a^	ND
		Sebacic acid	77.63 ± 2.51^a^	76.42 ± 1.23^a^	ND	ND
		Undecanedioic acid	85.64 ± 4.12^a^	86.86 ± 3.52^a^	ND	ND
	Lineolic acids	Corchorifatty acid F	4.29 ± 0.13^a^	3.79 ± 0.09^a^	ND	ND
Flavonoids	Flavonoid glycosides	Phlorizin	18.69 ± 0.04^a^	17.60 ± 0.30^a^	ND	ND
		Xanthine	6.94 ± 0.02^a^	6.48 ± 0.02^a^	ND	ND
Organooxygen	Alcohols and polyols	Panthothenic acid (Vit. B5)	8.70 ± 1.03^a^	10.65 ± 1.22^a^	ND	ND
		Quinic acid	29.36 ± 1.68^a^	28.33 ± 1.16^a^	70.71 ± 1.52^b^	74.31 ± 1.78^b^
		Shikimic acid	24.23 ± 0.07^a^	23.97 ± 0.92^a^	ND	ND
	Carbohydrates	Glycoprotein-phospho-D-hexose	2.42 ± 0.02^a^	2.60 ± 0.04^a^	73.62 ± 0.83^b^	89.40 ± 2.62^b^
Phenolic acids	Hydroxybenzoic acids	3-Hydroxybenzoic acid	5.91 ± 0.06^a^	4.76 ± 0.02^a^	45.86 ± 0.03^b^	105.28 ± 3.37^b^
		Protocatechuic acid	19.05 ± 1.63^a^	19.06 ± 0.61^a^	ND	ND
Phenols	Methoxyphenols	Homovanillic acid	99.88 ± 2.79^a^	100.53 ± 3.72^a^	ND	ND
Tannins	Hydrolyzable tannis	Ellagic acid	10.61 ± 0.08^a^	6.26 ± 0.75^a^	35.46 ± 0.32^b^	60.75 ± 1.62^c^

Different letters (^a^^,b,^^c^) in the same line means significant differences (*p* < 0.05).

ND, not determined.

Regarding the CS extract, a total of 24 compounds were presumptively annotated, with a greater abundance of lipids (6 compounds, representing 25%) and amino acids (5 compounds, representing 21%), followed by (poly)phenols (5 compounds, representing 21%), organic acids (4 compounds, representing 17%), alcohols and polyols (3 compounds, representing 12%), and carbohydrates (1 compound, representing 4%) (see Supplementary Table S3). Like the *in vitro* study, the compounds of interest permeated to a lesser extent for CS-loaded OFs than for the CS extract. A total of 11 compounds were annotated for the CS-loaded OFs, with amino acids being present in greater amounts (3 compounds, representing 27%), followed by (poly)phenols (2 compounds, representing 18%), lipids (2 compounds, representing 18%), organic acids (2 compounds, representing 18%), carbohydrates (1 compound, representing 9%), and alcohols and polyols (1 compound, representing 9%).

The amino acid L-tyrosine permeated the porcine buccal mucosa up to 66.76% and 135.84% at 30 min for the CS extract and CS-loaded OFs, respectively. This amino acid can be converted from phenylalanine (also identified in the CS extract) through hydroxylation, and is recognized for its antioxidant properties that protect the skin from the oxidative stress induced by UV radiation, supporting the skin's natural repair mechanism and promoting tissue regeneration (84). L-tyrosine is also involved in the synthesis of neurotransmitters such as dopamine and adrenaline, which play key roles in modulating inflammatory responses and promoting wound healing (84). Acetyl-L-leucine was another amino acid that permeated both samples. It is one of the main amino acids present in a commercialized formulation (Elental^®^) used for the treatment of 5-fluorouracil-induced mucositis (85). Notably, the cytotoxic effects of chemotherapeutic agents extend to salivary tissues, leading to diminished saliva volume and alterations in the salivary protein composition. These changes significantly contribute to the intensification of OM (85).

Ellagic acid was also identified, permeating 6.26% and 60.75% of the CS extract and CS-loaded OFs, respectively. This phenolic acid is well known for its antioxidant activity in lipid peroxidation and metal chelation, which safeguards cells against oxidative damage. It also shows chemopreventive, antimicrobial, anti-apoptotic, anti-inflammatory, and antimutagenic activities (86).

Overall, *in vitro* and *ex vivo* assays revealed similar qualitative trends in the permeation of key (poly)phenolic compounds and other bioactives from the CS-loaded orodispersible films and the free CS extract. However, some distinctions in the extent and profile of compound permeation were noted. As expected, lower absolute permeation percentages were observed in the *ex vivo* model using porcine buccal mucosa, likely due to the presence of a more complex biological barrier when compared to the *in vitro* TR146 cell model, which lacks tight junctions and may be more permissive to compound transport. Despite these differences in barrier properties, no unexpected changes in the identity or order of the permeating compounds emerged. In fact, several compounds such as isorhamnetin, homovanillic acid, and pyroglutamic acid, consistently demonstrated high permeation rates across both models. Interestingly, certain compounds, including L-tyrosine and ellagic acid, showed higher permeation in the *ex vivo* model from the CS-loaded OFs when compared to the free extract, which may be attributed to a more favorable microenvironment created by the film matrix or differences in compounds release kinetics. This suggests that the OFs not only preserved the permeation potential of the several bioactive compounds but may also enhance the mucosal absorption of specific molecules due to sustained hydration and increased mucosal contact time. Taken together, the *in vitro* and *ex vivo* findings support the effectiveness of the CS-loaded OFs as a viable buccal delivery system, capable of delivering relevant bioactive compounds across the oral mucosa. To the best of our knowledge, this is the first study that evaluated the potential of CS administered through OFs as active ingredient for the OM prevention using *in vitro* and *ex vivo* models.

Phenolic compounds often display limited oral (gastrointestinal) bioavailability due to the poor permeability and extensive first-pass metabolism. To address this, the present work targets buccal delivery, aiming a local action in the oral mucosa, while enabling transmucosal absorption that circumvents the first-pass metabolism. Consistent with this rationale, the *in vitro* TR146 model showed permeation of several CS-derived bioactives from the extract, including sebacic acid, isorhamnetin, epicatechin and homovanillic acid; CS-loaded OFs likewise delivered pyroglutamic acid, homovanillic acid, succinic acid, isorhamnetin, protocatechuic acid and caffeoylquinic acid across the model. In the *ex vivo* porcine buccal assay, the same qualitative profile was observed, with lower absolute percentages owing to the thicker biological barrier, while some compounds (e.g., L-tyrosine, ellagic acid) permeated more efficiently from the films than from the free extract, suggesting that the film matrix and the buccal residence can favor the uptake of specific molecules. Overall, these data indicate that a subset of low- to mid-molecular-weight phenolics and related acids in the CS are bioavailable via the buccal route, supporting both local therapeutic levels and potential systemic exposure, without gastrointestinal transit. Nevertheless, it should be emphasized that absolute systemic bioavailability was not determined in this study; *in vivo* pharmacokinetic and exposure–response studies are warranted.

Potential strategies to enhance the polyphenols release in future studies include the use of polymer blends, such as combining HPMC with more hydrophilic or mucoadhesive polymers like PVA or chitosan, to better modulate the film hydration and control the release profile. Additionally, the incorporation of release enhancers or permeation modulators, such as surfactants, bile salts, or natural terpenes, may facilitate the phenolic compounds diffusion from the polymeric matrix. These approaches have shown promising results in other mucoadhesive and buccal delivery systems for improving the release and mucosal uptake of bioactives. Such strategies are particularly relevant for polyphenolic compounds, which often display limited mobility due to their molecular complexity and potential interactions within the polymer network.

Generally, the incorporation of CS extract into orodispersible films resulted in a stable and functional delivery system with promising antioxidant, mucoadhesive, and permeation properties. Key bioactive compounds successfully permeated through *in vitro* and *ex vivo* buccal models, supporting the potential of this strategy for local delivery in OM prevention. Despite some reduction in mechanical properties and antioxidant activity when compared to the raw extract, the films maintained the structural integrity and biological relevance. These findings lay the groundwork for further preclinical studies and formulation refinements, particularly toward enhancing release kinetics and ensuring clinical translation of this natural-based therapeutic approach.

This study represents a preclinical proof of concept rather than a complete pharmacological evaluation. The findings support the buccal delivery of the selected phenolics, but further research is needed to confirm efficacy and safety *in vivo*. Future work will involve (i) testing in clinically relevant OM animal models to establish dose–response relationships and optimal dosing; (ii) standardization of CS extracts to key marker compounds (e.g., epicatechin, isorhamnetin) to ensure reproducibility; (iii) comparison of the extract with isolated compounds to determine contribution and potency; and (iv) comprehensive toxicological profiling to evaluate possible side effects at therapeutically relevant doses. These steps are essential to advance toward evidence-based development and potential regulatory approval.

## 4 Conclusion

This study demonstrated that CS extract can be successfully incorporated into OFs, yielding a stable and functional delivery system with antioxidant, mucoadhesive, and permeation properties relevant to OM prevention. Key phenolics, including epicatechin, protocatechuic acid, and isorhamnetin, permeated through *in vitro* and *ex vivo* models, supporting their potential for the local delivery at biologically meaningful concentrations. Although some reduction in antioxidant capacity and mechanical properties was observed when compared with the raw extract, the films maintained structural and functional integrity. The work represents a preclinical proof of concept, and further studies are needed to confirm the *in vivo* efficacy and safety, as previously mentioned. Importantly, the preparation method is relatively labor-intensive; therefore, future work should prioritize simplified and more sustainable production strategies that align with pharmaceutical development and green chemistry principles. Together, these steps will strengthen the evidence base and advance CS-loaded OFs toward clinical translation.

## Data Availability

The raw data supporting the conclusions of this article will be made available by the authors, without undue reservation.
