# Supplementary material for: Orodispersible films containing chestnut shell phenolics for buccal delivery: a preclinical approach for oral mucositis prevention
Source: Front Med Technol. 2025 Sep 18;7:1675082. doi: 10.3389/fmedt.2025.1675082 (PMC12488667; doi:10.3389/fmedt.2025.1675082)
Supplement: Supplementary file 1 [file Table1.docx]

**Supplementary Table S1 -** Putative annotation of compounds detected by LC-ESI-LTQ-Orbitrap-HRMS analysis regarding untargeted metabolomic profiling using MS-dial and MS-finder platforms.

| **Rt**  **(min)** | **Metabolite** | **Ontology** | **Neutral molecular formula** | **Average Mz** | **Theoretical mass** | **Error**  **(ppm)** | **Total score** | **MS^1^ isotopic spectrum** |
| --- | --- | --- | --- | --- | --- | --- | --- | --- |
| 0.76 | L-Malic acid | Hydroxy acid | C_4_H_6_O_5_ | 133.0155 | 134.0215 | -2.5373 | 7.23 | 134.01428:83116 135.01763:4074 136.02099:725 |
| 0.82 | Verbascose | Carbohydrate | C_30_H_52_O_26_ | 863.2420 | 863.2441 | 2.5381 | 6.81 | 863.26697:20574 864.27032:10099 865.27368:3349 |
| 1.14 | Fumaric acid | Dicarboxylic acid | C_4_H_4_O_4_ | 114.9631 | 115.0037 | 3.5164 | 6.67 | 115.00376:35380 116.00711:1547 117.01047:557 |
| 1.14 | Isocitric acid | Tricarboxylic acid | C_6_H_8_O_7_ | 191.0104 | 191.0202 | 4.1990 | 8.90 | 191.02008:1242036 192.02343:91598 193.02679:13890 |
| 1.25 | Pyroglutamic acid | Amino acid | C_5_H_7_NO_3_ | 128.0353 | 128.0355 | 2.1143 | 5.45 | 128.03522:67634 129.03857:4464 130.04193:1437 |
| 1.47 | Xanthine | Purine | C_5_H_4_N_4_O_2_ | 151.0254 | 152.1143 | 3.1420 | 6.67 | 151.02547:108327 152.02882:5681 153.03218:266 |
| 1.75 | Succinic acid | Dicarboxylic acid | C_4_H_6_O_4_ | 117.0191 | 117.0191 | 3.1679 | 5.55 | 117.01947:242833 118.02282:10538 119.02618:1505 |
| 1.78 | L-Tyrosine | Amino acid | C_9_H_11_NO_3_ | 180.0751 | 181.0738 | 1.5371 | 7.43 | 180.91753:11073 181.92088:574 182.92424:304 |
| 1.94 | Mevalonic acid | Fatty acid | C_6_H_12_O_4_ | 147.0774 | 148.0735 | 3.9641 | 6.84 | 148.98022:19821 149.98357:363 150.98693:0 |
| 2.23 | Gallic acid * | Benzoic acid | C_7_H_6_O_5_ | 169.0143 | 169.0143 | 4.3446 | 5.18 | 169.01428:83201 170.01763:5937 171.02099:822 |
| 2.50 | Adipic acid | Fatty acid | C_6_H_10_O_4_ | 145.0583 | 146.0579 | 2.9864 | 6.85 | 146.0872:17256 147.09055:1360 148.09391:0 |
| 3.28 | Myo-Inositol | Alcohols and polyols | C_6_H_12_O_6_ | 161.0444 | 161.0457 | 5.6848 | 5.50 | 161.04607:715644 162.04942:42718 163.05278:8059 |
| 3.47 | Quinic acid | Alcohols and polyols | C_7_H_12_O_6_ | 191.0663 | 192.0633 | 3.3551 | ND | 191.05621:10822 192.05956:626 193.06292:0 |
| 4.13 | Inosine | Carbohydrate | C_10_H_12_N_4_O_5_ | 267.0753 | 268.0807 | 4.6121 | ND | 268.80386:130934 269.80721:0 270.81057:344660 |
| 4.25 | Shikimic acid | Alcohols and polyols | C_7_H_10_O_5_ | 173.0483 | 174.0528 | 3.7482 | 7.84 | 174.08226:14201 175.08561:1232 176.08897:6558 |
| 4.37 | L-Phenylalanine | Amino acid | C_9_H_11_NO_2_ | 164.0751 | 165.0790 | 2.6254 | 8.26 | 165.05602:17963 166.05937:1566 167.06273:0 |
| 4.86 | Panthothenic acid (Vit. B5) | Alcohols and polyols | C_9_H_17_NO_5_ | 218.1034 | 218.1022 | 0.6427 | 5.37 | 218.10237:48219 219.10572:8398 220.10908:628 |
| 4.89 | Vescalagin | Hydrolyzable tannin | C_41_H_26_O_26_ | 466.0282 | 466.0290 | 3.5485 | 6.48 | 466.02896:63376 467.03231:11535 468.03567:412 |
| 4.95 | Pyrocatechol | Benzenoid | C_6_H_6_O_2_ | 108.0483 | 109.0295 | 0.0000 | 5.50 | 109.0295:112023 110.03285:8497 111.03621:531 |
| 5.05 | Neochlorogenic acid * | Alcohols and polyols | C_16_H_18_O_9_ | 352.0621 | 353.0882 | -0.0272 | 8.42 | 353.08817:193141 354.09152:53331 355.09488:6245 |
| 5.15 | L-Tryptophan | Amino acid | C_11_H_12_N_2_O_2_ | 203.0851 | 204.0899 | -0.2764 | ND | 204.08998:27546 205.08993:5310 206.0899:0 |
| 5.27 | Protocatechuic acid * | Hydroxybenzoic acid | C_7_H_6_O_4_ | 153.0196 | 153.0196 | 0.7685 | 5.28 | 153.01956:81820 154.02291:6771 155.02627:940 |
| 5.32 | 4-*O*-Caffeoylquinic acid * | Hydroxycinnamic acid | C_16_H_18_O_9_ | 353.0885 | 353.0885 | -3.6764 | 8.48 | 353.08853:188163 354.09188:53844 355.09524:7459 |
| 5.34 | Methylgallate * | Benzoic acid | C_8_H_8_O_5_ | 182.0352 | 183.0306 | 0.0000 | 7.02 | 183.03064:819017 184.03399:100662 185.03735:6341 |
| 5.40 | Acetyl-L-Leucine | Amino acid | C_8_H_15_NO_3_ | 172.1515 | 173.1052 | 1.5172 | 8.34 | 173.10226:14201 174.10561:1232 175.10897:6558 |
| 5.40 | Homovanillic acid | Methoxyphenol | C_9_H_10_O_4_ | 180.0725 | 181.0512 | 0.0000 | 8.28 | 181.05124:447078 182.05459:63292 183.05795:4723 |
| 5.51 | Epicatechin * | Flavonoid | C_15_H_14_O_6_ | 289.0719 | 289.0724 | -1.9624 | 7.77 | 289.07242:294892 290.07577:70716 291.07913:5734 |
| 5.54 | Glycoprotein-phospho-D-hexose | Carbohydrate | C_6_H_12_O_6_ | 179.0549 | 180.0633 | 2.5163 | 6.64 | 179.05487:23781 180.05822:1424 181.06158:44357 |
| 5.55 | Pimelic acid | Fatty acid | C_7_H_12_O_4_ | 159.0754 | 160.0736 | 1.8564 | 6.72 | 159.06566:45785 160.06901:5759 161.07237:332 |
| 5.56 | 3-Hydroxybenzoic acid * | Benzoic acid | C_7_H_6_O_3_ | 137.0246 | 137.0246 | 1.7398 | 5.56 | 137.02458:87910 138.02793:7344 139.03129:668 |
| 5.61 | Epigallocatechin gallate * | Flavanoid | C_22_H_18_O_11_ | 457.0851 | 458.0849 | 3.4682 | 6.62 | 457.07568:34102 458.07903:12858 459.08239:2246 |
| 5.62 | 2,6-Dihydroxybenzoic acid * | Benzoic acid | C_7_H_6_O_4_ | 153.0251 | 154.0266 | 1.1795 | 7.35 | 153.02629:22900 154.02964:1347 155.033:106 |
| 5.67 | m-Cresol | Phenol | C_7_H_8_O | 107.0563 | 108.0575 | 0.5316 | ND | 107.05032:32272 108.05367:2969 109.05703:0 |
| 5.72 | Rutin * | Flavonoid glycoside | C_27_H_30_O_16_ | 609.1463 | 609.1474 | 2.2707 | 8.19 | 609.1474:220069 610.15075:101117 611.15411:23019 |
| 5.80 | Quercetin 3-*O*-galactoside * | Flavonoid glycoside | C_21_H_20_O_12_ | 463.0891 | 463.0891 | 1.7539 | 8.38 | 463.08905:299453 464.0924:107861 465.09576:20583 |
| 5.82 | Hydroxycinnamic acid isomer I | Hydroxycinnamic acid | C_9_H_8_O_3_ | 163.0404 | 163.0404 | 0.6326 | 5.25 | 163.04042:100914 164.04377:14967 165.04713:455 |
| 5.86 | Vanillin * | Methoxyphenol | C_8_H_8_O_3_ | 151.0402 | 151.0402 | 0.8521 | 5.43 | 151.04024:55671 152.04359:4944 153.04695:342 |
| 5.87 | Ellagic acid * | Benzoic acid | C_14_H_6_O_8_ | 301.0051 | 302.0047 | -2.6271 | 7.45 | 300.99747:267836 302.00082:67566 303.00418:7245 |
| 5.88 | Homoveratric acid | Methoxybenzene | C_10_H_12_O_4_ | 195.0669 | 195.0669 | 3.2409 | 6.19 | 195.06694:85437 196.07029:15019 197.07365:1141 |
| 5.93 | Suberic acid | Fatty acid | C_8_H_14_O_4_ | 173.0851 | 174.0892 | 1.6865 | 8.62 | 173.08127:79674 174.08462:14713 175.08798:722 |
| 5.95 | Dihydroferulic acid * | Hydoxycinnamic acid | C_10_H_12_O_4_ | 195.0762 | 196.0735 | -1.6278 | 5.95 | 195.06584:19615 196.06919:3085 197.07255:240 |
| 6.00 | Naringin * | Flavonoid glycoside | C_27_H_32_O_14_ | 579.1713 | 579.1732 | 2.9924 | 7.42 | 579.17316:293758 580.17651:135313 581.17987:28635 |
| 6.01 | Sinapic acid * | Hydroxycinnamic acid | C_11_H_12_O_5_ | 223.0662 | 224.0684 | 4.2162 | 6.67 | 223.06075:25010 224.0641:5722 225.06746:349 |
| 6.14 | Hydroxycinnamic acid Isomer II | Hydroxycinnamic acid | C_9_H_8_O_3_ | 163.0462 | 164.0473 | 1.3961 | 5.26 | 163.04269:68213 164.04604:7591 165.0494:4847 |
| 6.22 | Secoisolariciresinol * | Lignans | C_20_H_26_O_6_ | 361.1655 | 361.1663 | -1.9736 | 7.11 | 361.16629:162284 362.16964:58709 363.173:6237 |
| 6.26 | Phlorizin * | Flavonoid *O*-glycoside | C_21_H_24_O_10_ | 435.1363 | 436.1369 | 4.7261 | 8.46 | 435.13037:300575 436.13372:94283 437.13708:15243 |
| 6.55 | Sebacic acid | Fatty acid | C_10_H_18_O_4_ | 201.1137 | 201.1137 | 0.0063 | 7.03 | 201.11372:104834 202.11707:15341 203.12043:1531 |
| 6.61 | Luteolin * | Flavonoid | C_15_H_10_O_6_ | 285.0461 | 286.0477 | 1.5265 | ND | 285.03928:136912 286.04263:38657 287.04599:2821 |
| 6.79 | Sativic acid | Fatty acid | C_18_H_36_O_6_ | 347.2439 | 347.2446 | 2.2134 | 6.90 | 347.24457:279813 348.24792:79447 349.25128:8059 |
| 6.89 | Apigenin * | Flavonoid | C_15_H_20_O_5_ | 269.0461 | 270.0528 | 3.6282 | 6.89 | 269.04443:113259 270.04778:32998 271.05114:1976 |
| 6.93 | Undecanedioic acid | Fatty acid | C_11_H_20_O_4_ | 215.1361 | 216.1362 | -2.5168 | 6.93 | 215.12799:80355 216.13134:20670 217.1347:1032 |
| 6.94 | Isorhamnetin * | Flavonoid | C_16_H_12_O_7_ | 315.0510 | 315.0517 | 2.2463 | 7.38 | 315.0517:956562 316.05505:195939 317.05841:17997 |
| 7.36 | Corchorifatty acid F | Lineolic acid | C_18_H_32_O_5_ | 327.2291 | 328.2250 | 3.9941 | 5.83 | 327.21793:17969 328.22128:4306 329.22464:3498 |
| 7.89 | Sakuranin | Flavonoid glycoside | C_23_H_20_N_4_O_6_ | 447.1361 | 448.1382 | 2.5174 | 7.97 | 447.13425:10526 448.1376:2979 449.14096:0 |
| 8.23 | Crocetin | Curcuminoid | C_20_H_24_O_4_ | 327.1641 | 328.1675 | 4.7516 | 5.78 | 327.16159:64446 328.16494:13030 329.1683:3106 |
| 8.49 | Colnelenic acid | Fatty acid | C_18_H_28_O_3_ | 291.2526 | 292.2038 | 3.6283 | 5.58 | 291.19958:49921 292.20293:6968 293.20629:2432 |
| 8.80 | Herbacetin | Flavonoid glycoside | C_28_H_30_O_17_ | 637.1474 | 638.1483 | 2.5179 | 8.80 | 637.14056:115806 638.14391:49807 639.14727:4868 |
| 9.39 | Ajmaline | Alkaloid | C_20_H_26_N_2_O_2_ | 325.2763 | 326.1994 | 0.2774 | 5.37 | 325.18454:398558 326.18789:75607 327.19125:14636 |
| 12.07 | Auraptene | Prenol lipid | C_16_H_26_O_3_S | 297.1583 | 298.1602 | 2.7356 | 6.32 | 297.15106:72262 298.15441:12607 299.15777:2636 |
| 13.82 | Hydroxyadipic acid | Fatty acid | C_6_H_10_O_5_ | 161.0457 | 161.0456 | -1.4679 | 8.66 | 161.04562:32251 162.04897:4976 163.05233:1430 |

**Supplementary Table S2 –** Quantification of permeated compounds of chestnut shells (CS) extract and orodispersible films (OFs) incorporated with CS extract through the in vitro buccal model (TR146 cells) (n=3). Results are expressed as mean ± SD. Different letters in the same line means significant differences between samples (p < 0.05).

| **Rt (min)** | **[m-H]^-^ (*m*/*z*)** | **Compound/Metabolite** | **Quantification** | |  |
| --- | --- | --- | --- | --- | --- |
|  |  |  | **CS extract (mg/ml)** | **CS-loaded OFs (mg/g film)** |  |
| 0.82 | 863.2420 | Verbascose | 36.03 ± 1.04^a^ | 80.50 ± 3.96^b^ |  |
| 1.14 | 191.0104 | Isocitric acid | 222.23 ± 6.57^a^ | 306.36 ± 13.65^b^ |  |
| 1.25 | 128.0353 | Pyroglutamic acid | 41.32 ± 2.63^b^ | 28.93 ± 1.88^a^ |  |
| 1.75 | 117.0191 | Succinic acid | 1.60 ± 0.03^a^ | 0.82 ± 0.01^a^ |  |
| 3.28 | 161.0444 | Myo-Inositol | 0.54 ± 0.01^a^ | 0.42 ± 0.01^a^ |  |
| 4.95 | 108.0483 | Pyrocatechol | 0.46 ± 0.01^a^ | 0.36 ± 0.02^a^ |  |
| 5.05 | 352.0621 | Neochlorogenic acid | 0.48 ± 0.05^a^ | 0.36 ± 0.02^a^ |  |
| 5.27 | 153.0196 | Protocatechuic acid | 0.53 ± 0.03^a^ | 0.41 ± 0.02^a^ |  |
| 5.32 | 353.0885 | Caffeoyl quinic acid | 0.40 ± 0.02^a^ | 0.38 ± 0.03^a^ |  |
| 5.34 | 182.0352 | Methyl gallate | 0.37 ± 0.01^a^ | 0.37 ± 0.03^a^ |  |
| 5.40 | 180.0725 | Homovanillic acid | 0.58 ± 0.03^a^ | 0.41 ± 0.01^a^ |  |
| 5.51 | 289.0719 | Epicatechin | 0.41 ± 0.03^a^ | 0.36 ± 0.02^a^ |  |
| 5.56 | 137.0246 | 4-Hydroxybenzoic acid | 1.52 ± 0.02^a^ | 0.36 ± 0.03^a^ |  |
| 5.80 | 463.0891 | Quercetin 3-galactoside | 0.36 ± 0.01^a^ | 0.36 ± 0.01^a^ |  |
| 5.86 | | 151.0402 | Vanillin | 0.70 ± 0.02^a^ | 0.44 ± 0.05^a^ |
| 6.22 | 361.1655 | Secoisolariciresinol | 0.43 ± 0.02^a^ | 0.37 ± 0.01^a^ |  |
| 6.55 | 201.1137 | Sebacic acid | 26.39 ± 1.68^b^ | 10.60 ± 0.96^a^ |  |
| 6.79 | 347.2439 | Sativic acid | 66.13 ± 3.84^b^ | 39.85 ± 2.53^a^ |  |
| 6.94 | 315.0510 | Isorhamnetin | 0.38 ± 0.04^a^ | 0.38 ± 0.01^a^ |  |
| 13.82 | 161.0457 | 2-Hydroxyadipic acid | 0.51 ± 0.02^a^ | 0.43 ± 0.03^a^ |  |

**Supplementary Table S3 –** Quantification of permeated compounds of chestnut shells (CS) extract and orodispersible films (OFs) incorporated with CS extract through porcine buccal mucosa (Franz cells) (n=3). Results are expressed as mean ± SD. Different letters in the same line means significant differences between samples (p < 0.05).

| **Rt (min)** | **[m-H]^-^ (*m*/*z*)** | **Compound/Metabolite** | **Quantification** | |
| --- | --- | --- | --- | --- |
|  |  |  | **CS extract (mg/ml)** | **CS-loaded OFs (mg/g film)** |
| 1.14 | 114.9631 | Fumaric acid | 4.47 ± 0.98^b^ | 0.40 ± 0.01^a^ |
| 1.14 | 191.0104 | Isocitric acid | 2.20 ± 0.05^a^ | 2.21 ± 0.16^a^ |
| 1.47 | 151.0254 | Xanthine | 25.47 ± 0.13^b^ | 1.16 ± 0.02^a^ |
| 1.75 | 117.0191 | Succinic acid | 1.26 ± 0.01^b^ | 0.38 ± 0.01^a^ |
| 1.78 | 180.0751 | L-Tyrosine | 9.02 ± 0.38^b^ | 0.99 ± 0.03^a^ |
| 1.94 | 147.0774 | Mevalonic acid | 94.49 ± 4.52^b^ | 7.60 ± 1.00^a^ |
| 2.50 | 145.0583 | Adipic acid | 93.48 ± 2.39^b^ | 11.84 ± 2.11^a^ |
| 3.47 | 191.0663 | Quinic acid | 1.02 ± 0.02^b^ | 0.39 ± 0.01^a^ |
| 4.25 | 173.0483 | Shikimic acid | 1.59 ± 0.01^b^ | 0.27 ± 0.02^a^ |
| 4.37 | 164.0751 | L-Phenylalanine | 5.52 ± 0.24^b^ | 0.42 ± 0.01^a^ |
| 4.86 | 218.1034 | Panthothenic acid (Vit. B5) | 20.13 ± 1.64^b^ | 0.89 ± 0.05^a^ |
| 5.15 | 203.0851 | L-Tryptophan | 4.18 ± 0.26^a^ | 1.18 ± 0.09^a^ |
| 5.27 | 153.0196 | Protocatechuic acid | 0.97 ± 0.03^b^ | 0.13 ± 0.01^a^ |
| 5.40 | 172.1515 | N-Acetyl-L-leucine | 9.12 ± 0.17^b^ | 0.99 ± 0.01^a^ |
| 5.40 | 180.0725 | Homovanillic acid | 2.03 ± 0.03^a^ | 1.67 ± 0.03^a^ |
| 5.54 | 179.0549 | Glycoprotein-phospho-D-mannose | 22.71 ± 1.95^b^ | 3.11 ± 0.05^a^ |
| 5.55 | 159.0754 | Pimelic acid | 26.17 ± 2.25^a^ | 30.41 ± 0.01^a^ |
| 5.56 | 137.0246 | 4-Hydroxybenzoic acid | 1.18 ± 0.1^b^ | 0.09 ± 0.02^a^ |
| 5.87 | 301.0051 | Ellagic acid | 7.15 ± 2.50^b^ | 1.27 ± 0.05^a^ |
| 6.14 | 163.0462 | 2-Hydroxycinnamic acid | 0.49 ± 1.28^a^ | 0.13 ± 0.02^a^ |
| 6.26 | 435.1363 | Phlorizin | 1.93± 0.06^a^ | 0.87 ± 0.06^a^ |
| 6.55 | 201.1137 | Sebacic acid | 38.01± 3.41^a^ | 32.46 ± 3.54^a^ |
| 6.93 | 215.1361 | Undecanedioic acid | 23.93 ± 1.12^a^ | 23.67 ± 5.00^a^ |
| 7.36 | 327.2291 | Corchorifatty acid F | 32.51 ± 0.74^b^ | 0.85 ± 0.01^a^ |
